# Reproducibility for Heart Rate Variability Analysis during 6-Min Walk Test in Patients with Heart Failure and Agreement between Devices

**DOI:** 10.1371/journal.pone.0167407

**Published:** 2016-12-09

**Authors:** Lays Magalhães Braga, Gustavo Faibischew Prado, Iracema Ioco Kikuchi Umeda, Tatiana Satie Kawauchi, Adriana Marques Fróes Taboada, Raymundo Soares Azevedo, Horacio Gomes Pereira Filho, César José Grupi, Hayala Cristina Cavenague Souza, Dalmo Antônio Ribeiro Moreira, Naomi Kondo Nakagawa

**Affiliations:** 1 Department of Physiotherapy, Faculdade de Medicina da Universidade de São Paulo, São Paulo, Brazil; 2 Pulmonary Division of Heart Institute (InCor) do Hospital das Clínicas da Faculdade de Medicina da Universidade de São Paulo, São Paulo, Brazil; 3 Dante Pazzanese Institute of Cardiology São Paulo State, São Paulo, Brazil; 4 Department of Pathology, University of São Paulo Medical School, São Paulo, Brazil; 5 Cardiology Division of Heart Institute (InCor) do Hospital das Clínicas da Faculdade de Medicina da Universidade de São Paulo, São Paulo, Brazil; University of Minnesota, UNITED STATES

## Abstract

Heart rate variability (HRV) analysis is a useful method to assess abnormal functioning in the autonomic nervous system and to predict cardiac events in patients with heart failure (HF). HRV measurements with heart rate monitors have been validated with an electrocardiograph in healthy subjects but not in patients with HF. We explored the reproducibility of HRV in two consecutive six-minute walk tests (6MW), 60-minute apart, using a heart rate monitor (PolarS810i) and a portable electrocardiograph (called Holter) in 50 HF patients (mean age 59 years, NYHA II, left ventricular ejection fraction ~35%). The reproducibility for each device was analysed using a paired t-test or the Wilcoxon signed-rank test. Additionally, we assessed the agreement between the two devices based on the HRV indices at rest, during the 6MW and during recovery using concordance correlation coefficients (CCC), 95% confidence intervals and Bland-Altman plots. The test-retest for the HRV analyses was reproducible using Holter and PolarS810i at rest but not during recovery. In the second 6MW, patients showed significant increases in rMSSD and walking distance. The PolarS810i measurements had remarkably high concordance correlation [0.86<CCC<0.99] based on Holter at rest, during 6MW and recovery. At higher rates, a small effect in increasing differences between Holter and Polar in R-R intervals was observed. In conclusion, our study showed good reproducibility of HRV at rest in two consecutive 6MW using Holter and PolarS810i. Additionally, PolarS810i produced good agreements in short-term HRV indices based on Holter simultaneous recordings at rest, during the 6MW and recovery in HF patients.

## Introduction

In recent decades, studies have demonstrated that beat-to-beat interval variation analysis, called heart rate variability (HRV), is a useful non-invasive method to assess the autonomic modulation of the sinus node. HRV describes the dispersion of R-R intervals in determined temporal series that reflect the balance between the dynamic interferences of the sympathetic and parasympathetic nervous systems with positive and negative chronotropic effects, respectively. HRV analysis derived from an 24-hour electrocardiogram (ECG) has shown stability in healthy subjects [1], in patients with heart failure (HF) New York Heart Association (NYHA) functional class II-III taking their usual medications [2], or in patients with congestive HF secondary to coronary artery disease [3].

Studies on patients with HF have shown associations between reduced HRV and sudden death [4], one-year mortality [5] and overall mortality [6] using a 24-hour ECG. Using a short-term ECG, studies have shown associations between HRV reduction and an increased hazard ratio of new HF in hypertensive subjects [7] and sudden death in patients with HF [8]. Increased HRV was shown to have a protective role in the prevention of cardiac events [9]. Heart rate monitors are inexpensive and make it moderately simple to record, edit and analyse R-R intervals and HRV indices. In healthy subjects, the reproducibility of short-term HRV analysis from heart rate monitors based on ECGs has been extensively investigated, and its ability has been shown to be comparable to portable ECGs [10–17]. However, it is of interest to evaluate whether there is agreement between short-term HRV analysis from heart rate monitors and ECGs in patients with HF.

To our knowledge, the reproducibility of short-term HRV analysis using portable ECGs and heart rate monitors and the agreement between heart rate monitors and ECGs, based on portable ECGs have not been investigated in patients with HF. We hypothesized that the two devices may have a similar ability to reproduce HRV indices in patients with moderate HF. Good reproducibility and agreement between devices are essential for the maximal utility of instruments in clinical follow-up and research. Due to the limited information available, we investigated the reproducibility of short-term HRV using simultaneously a portable ECG (Holter Cardiolight, Cardios Ind., São Paulo, Brazil) and a heart rate monitor (PolarS810i, Polar Electro-OY, Kempele, Finland) at rest and during recovery from two consecutive 6MW, 60-min apart. We also investigated the agreement of short-term HRV indices from Polar S810i based on Holter at rest, during the six-min walk test (6MW) and during recovery in stable patients with moderate HF.

## Methods

### Participants

This study followed the ethical guidelines of the Declaration of Helsinki, and it was approved by the Ethical Committees of University of Sao Paulo School of Medicine and Dante Pazzanese Heart Institute of Sao Paulo State (CEP 232/12). The patients entered into the study after providing their writing informed consent.

Consecutive patients with HF, NYHA functional class II with a left ventricular ejection fraction (LVEF) < 40% were recruited from a list of a cardiac rehabilitation centre in a six-month period in 2014. Exclusion criteria were uncontrolled arrhythmia, chronic atrial fibrillation, acute HF, decompensated HF, ectopic beats in ≥ 5% of measurements, the use of a paced rhythm, current smoking or a smoking time cessation < 24 months and any orthopaedic or cognitive problems that could affect 6MW performance.

The subjects’ weight and height were used to calculate their body mass index. Hypertension, diabetes and smoking history were recorded. Hypertension was defined according to the American Association of Cardiology [18]. Diabetes was defined in accordance with the American Association of Diabetes [19]. Medications routinely used by the patients were recorded. Subjects self-reported their regular physical activity during the week prior to the study using the International Physical Activity Questionnaire validated for the Brazilian population [20]. Their answers allowed them to be classified as “active” (30 min of regular physical activity ≥ 3 times per week) or “sedentary”.

All subjects reported to the laboratory between 8 and 11 A.M. on one single occasion to perform two consecutive 6MW, 60-min apart. Holter and Polar S810i were used simultaneously. Subjects were instructed to eat a light breakfast at least 2 hours before testing, to take their usual medications, to abstain from consuming tea, alcohol and beverages containing caffeine, and to refrain from heavy physical exertion in the 24 hours preceding the assessments.

**Fig 1:**
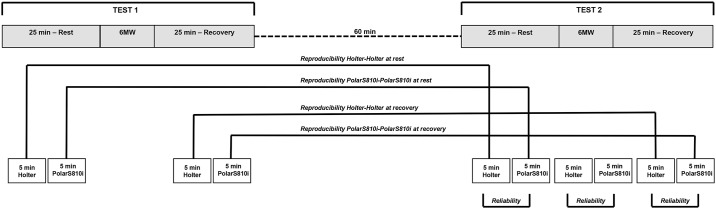
The study design shows the two consecutive 6MW to analyse the reproducibility of the two devices, Holter and PolarS810i at rest and during recovery and the reliability of PolarS810i based on Holter at rest, 6MW and during recovery from the second 6MW.

Subjects underwent a physical examination in a controlled environment (24°C room temperature and 55–60% relative humidity) and stayed at supine rest for 25 min before and after the two 6MW. The two 6MW were performed in accordance with the ATS guidelines [21]. For the reproducibility of the test-retest 6MW, R-R intervals were recorded and the HRV data were analysed at rest and during recovery from the two 6MW using both devices, Holter and PolarS810i. For the HRV analysis of agreement between PolarS810i and holter, based on Holter, we used data from the second 6MW (Fig 1).

### Heart rate variability using Holter and Polar S810i

Subjects underwent a simultaneous 60-min R-R recording using Holter and PolarS810i for each 6MW. In brief, subjects were prepared for the attachment of a 3-lead ECG (called Holter, CardioLight model, Cardios Inc., São Paulo, Brazil) that was in a standard configuration and placed on the sternum manubrium, at the xiphoid appendix and at the 5^th^ intercostal space in the middle clavicular line. Then, a strap of the PolarS810i was fixed around the chest of the subject in accordance with the manufacturer’s instructions. Subjects were instructed to rest in supine with their head supported by a pillow and not to talk or move excessively any part of their body. R-R recordings were done when the subjects were resting in supine at baseline, walking for 6 min and recovering from the 6MW. In each phase of the 6MW, only a 5 min time series with a minimum of 256 R-R intervals was analysed for HRV indices, as recommended [1]. At rest, the 5-min right before beginning the 6MW was taken to limit possible influence of equipment installation, talking and deambulation (the washout effect). At 6-minute period of the 6MW, the first minute was excluded to get more stable and comparable signals. At recovery, the first minutes from the total 25-minute were taken as the time-series for comparisons. All assessors were blinded to subjects’ identification and conditions.

ECG data acquisition was performed using a Holter device at a sampling frequency of 800 Hz with temporal resolution of 1 ms for each R-R interval. Data were stored in a computer and further analysed by two physicians using CardioSmart S550 Software (Cardios Inc., São Paulo, Brazil). Each ECG recording was inspected for ectopic beats and artefacts, which were replaced with the mean value of the previous and subsequent intervals with the accepted R-R interval in accordance with the recommendations of the Task Force of the European Society of Cardiology and the North American Society of Pacing and Electrophysiology [1].

The R-R intervals of the Polar S810i registered throughout the study were downloaded and edited on a laptop computer with the aid of software (Polar Pro-Trainer 5 Software, Kempele, Finland). A sampling frequency of 1,000 Hz with temporal resolution of 1-ms for each R-R interval was chosen. The Polar software has an automatic R-R interval filtering that removes detected errors and aberrant beats and interpolates intervals with the aid of an algorithm described above. After this, all registrations were visually inspected for removal of artifacts or aberrant beats within the limit of 5% of the R-R intervals. Data were analysed by a physiotherapist and a physician using Kubios HRV Analysis Software 2.1 (Biomedical Signal Analysis Group, Kupio, Finland). For the analysis of agreement between the Polar and Holter for the data acquisition, R-R intervals registrations were tested. For the time domain, HRV analysis included the SDNN (standard deviation of all R-R intervals), the root mean square successive difference between R-R intervals (rMSSD), and the percentage of successive differences in the R-R interval of which the absolute values exceeds 50-ms (pNN50). For the frequency-domain, spectral analysis was calculated using fast Fourier transform algorithms to analyse LF in normalized units (LF nu), the high frequency in normalized units (HF nu) and the LF/HF ratio.

### Statistical analysis

Statistical analyses were conducted using SPSS statistical software version 19.0 (SPSS Inc., IL, USA) and R 3.2.1 software. Data are summarized as means, standard deviations and proportional frequencies, when appropriate.

The reproducibility of the HRV indices (R-R intervals, SDNN, rMSSD, pNN50, LF nu, HF nu and LF/HF ratio) and the walking distance of the test-retest 6MW using Holter and Polar S810i were compared using a paired t-test or a Wilcoxon signed-rank test. A p-value < 0.05 was considered statistically significant.

The agreement of the HRV analysis with between PolarS810i and Holter, based on Holter was tested in the second 6MW in HF patients (at rest, during the 6MW and during recovery) and was estimated using the Concordance Correlation Coefficient (CCC) with 95% confidence intervals (95% CI) [22], and Bland-Altman plots [23].

Linear regression models were developed to explore the possibility of biases of proportionality adopting the average value of Holter and Polar measures as independent variables and the differences between both measures as dependent variables.

## Results

Fifty-four patients with mild/moderate systolic dysfunction (17% ≤ LVEF ≤ 55%) were admitted to the study and underwent two consecutive 6MW, 60-minute apart. Holter and Polar S810i were used simultaneously. The recordings of four patients were excluded due to aberrant beats/artefacts ≥ 5% after editing. Demographic and clinical data (Table 1) showed that the majority of patients were older, male, hypertensive and sedentary and had ischaemic heart disease.

**Table 1:** Demographic and clinical data of 50 patients with HF NYHA class II are presented as mean values (SD) or absolute numbers and proportion of patients when appropriate.

	HF subjects n = 50
**Age**, years	59 (6.2)
**BMI**, kg/m^2^	24.6 (5.2)
**Male**, n (%)	39 (78)
**LVEF**, %	35.4 (7.8)
**Etiology**, n (%)	
Ischemic	38 (76)
Non-ischemic	12 (24)
**Other morbidities**, n (%)	
Hypertension	50 (100)
Diabetes	21 (42)
Myocardial infarction	38 (76)
**Medications**, n (%)	
Antiarrhythmics	9 (18)
Anticoagulants	8 (16)
Angiotensin receptor blockers	12 (24)
ACE inibitors	31 (62)
Antiplatelet agents	45 (90)
Beta-blockers	49 (98)
Digitalics	13 (26)
Diuretics	49 (98)
Antidepressants	27 (54)
**Arrhythmias > 10 beats/hour**, n (%)	
Atrial premature beats	9 (18)
Ventricular premature beats	19 (38)
**Smoking**	
Time of cessation, years	11 (6)
Pack-years	22.8 (14.7)
**Physical activity**, n patients (%)	
Sedentary	38 (76)
Active	12 (24)

Abbreviations: HF, heart failure, NYHA, New York Heart Association, BMI, body mass index, LVEF, left ventricular ejection fraction, ACE, angiotensin-converting enzyme

The walking distance was higher in the second 6MW (489.2 ± 64.7 m) than the first 6MW (474.1 ± 65.8 m, p < 0.001).

The reproducibility of R-R intervals was first analysed. Similar mean values were shown in the test-retest 6MW at rest and during recovery when using Holter (p = 0.938 and p = 0.714, respectively) and PolarS810i (p = 0.919 and p = 0.745, respectively). The HRV indices using both devices also showed similar mean values at rest and during recovery (Table 2). The rMSSD data showed a slightly significant difference between the two 6MW in the recovery phase using both devices.

**Table 2:** Reproducibility of HRV analysis by Holter and by PolarS810i at rest and during recovery from the 6MW. Data are presented as mean (min-max) values.

	Rest			Recovery		
	Test 1	Test 2	1x2 p-value	Test 1	Test 2	1x2 p-value
**Holter**						
HR,bpm	63 (50–96)	62 (48–95)		67 (50–110)	66 (50–109)	
SDNN, ms	29.9 (13–56)	29.3 (9–57)	.591	29.7 (11–51)	31.0 (14–55)	.306
rMSSD, ms	16.8 (5–35)	17.2 (8–30)	.352	15.9 (7–32)	17.3 (8–34)	.020
pNN50, %	1.3 (0–7)	1.4 (0–8)	.394	1.1 (0–7)	1.1 (0–5)	.766
LF nu	73.4 (54–100)	72.2 (25–93)	.696	72.0 (46–93)	70.5 (16–100)	.904
HF nu	28.9 (9–49)	27.3 (8–49)	.540	28.5 (9–58)	28.1 (5–57)	.915
LF/HF	3.4 (1–11)	3.6 (1–12)	.697	3.3 (1–9)	3.6 (1–19)	.569
**PolarS810i**						
HR,bpm	62 (50–94)	62 (47–95)		66 (49–109)	66(49–110)	
SDNN, ms	28.4 (12–53)	27.4 (9–55)	.505	28.0 (10–49)	29.4 (54–12)	.295
rMSSD, ms	15.2 (4–33)	15.8 (6–28)	.338	14.3 (6–31)	15.7 (6–31)	.025
pNN50, %	1.0 (0–6)	1.0 (0–7)	.871	0.7 (0–6)	0.8 (0–4)	.715
LF nu	72.2 (51–98)	73.1 (22–94)	.337	71.7 (42–94)	72.0 (17–95)	.412
HF nu	28.7 (7–48)	27.2 (8–50)	.429	28.0 (9–51)	27.8 (5–53)	.866
LF/HF	3.5 (1–14)	3.6 (1–12)	.418	3.3 (1–10)	3.8 (1–20)	.601

Abbreviations: HR, heart rate, SDNN, standard deviation of all R-R intervals, rMSSD, root mean square successive difference between R-R intervals, and pNN50, percentage of successive differences in the R-R interval of which the absolute values exceeds 50-ms, LF nu, low-frequency normalized unit, HF nu, high-frequency normalized unit, LF/HF, low and high-frequency ratio, 6MW, six-min walk test

**Fig 2:**
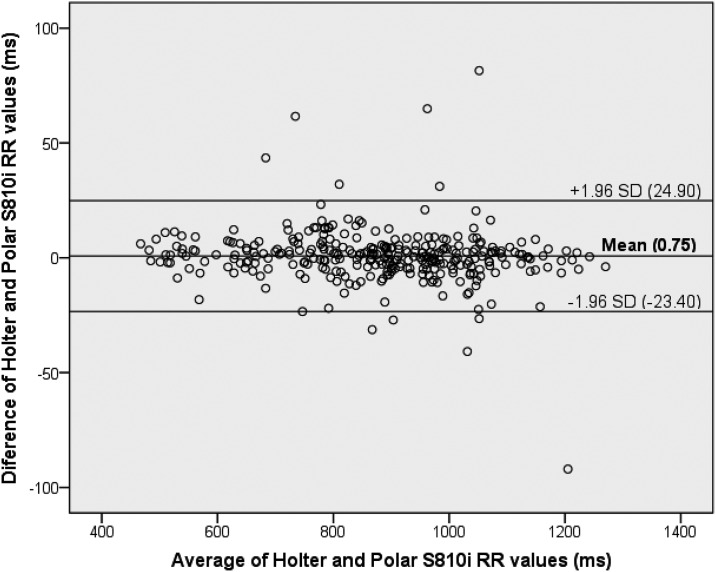
Reliability between Holter and PolarS810i for R-R intervals at rest and movement using Bland-Altman plots.

The agreement between crude data of R-R intervals (Fig 2) was high (CCC = 0.999, p<0.001) with lower dispersion beyond the limits (± 1.96 SD) from the Bland-Altman diagram.

**Fig 3:**
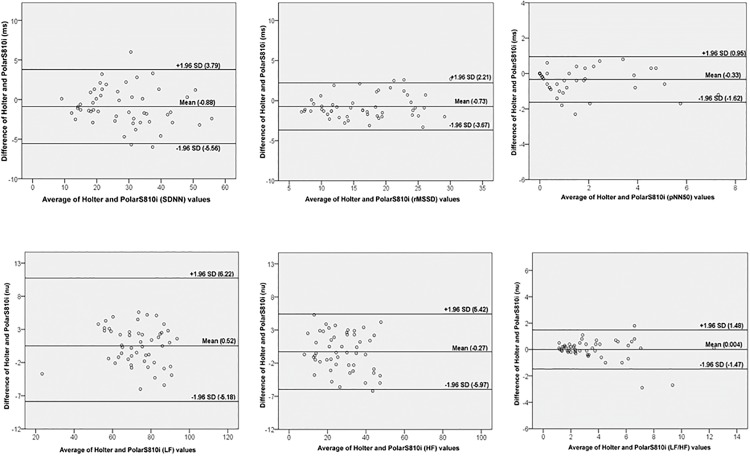
Reliability between Holter and Polar S810i for HRV analyses at rest using Bland-Altman plots.

There was a small but significant effect (β = -0.010) of the independent variable (the average R-R intervals between Holter and PolarS810i) on the differences between measures obtained with both, Holter and Polar (Table 3). The other regression models proved to be not statistically significant, removing the possibility of any biases of proportionality. Bland-Altman plots showed good agreement between the Holter and PolarS810i in time and frequency domains that were assessed at rest (Fig 3). PolarS810i showed good predictive performance to assess Holter HRV values in different ranges of variation of variables. During the 6MW and recovery, the CCC between values from Holter and Polar S810i showed good agreements for most HRV indices (Table 4).

**Table 3:** Linear regression models for proportionality bias in absolute differences and average (a-) values between Holter and Polar810i.

	Constant	β	95% CI	p-value
**a-RR**	9.255	-0.010	-0.018; -0.002	0.016
**a-SDNN**	0.086	-0.034	-0.095; 0.026	0.262
**a-rMSSD**	-1.516	0.047	-0.021; 0.115	0.171
**a-pNN50**	-0.252	-0.065	-0.171; 0.041	0.224
**a-LF**	1.898	-0.019	-0.083; 0.046	0.558
**a-HF**	1.142	-0.051	-0.125; 0.022	0.168
**a-LF/HF**	0.281	-0.079	-0.169; 0.012	0.086

Abbreviations: RR, intervals between two peaks, SDNN, standard deviation of all R-R intervals, rMSSD, root mean square successive difference between R-R intervals, and pNN50, percentage of successive differences in the R-R interval of which the absolute values exceeds 50-ms, LF nu, low-frequency normalized unit, HF nu, high-frequency normalized unit, LF/HF, low and high-frequency ratio, β, linear regression coefficient

**Table 4:** Concordance Correlation Coefficients [95% CI] of HRV indices between Holter and PolarS810i at rest, during the 6MW and recovery.

	Rest	6MW	Recovery
**R-R, ms**	0.99 [0.98–0.99]	0.99 [0.97–0.99]	0.99 [0.98–0.99]
**SDNN, ms**	0.97 [0.96–0.98]	0.95 [0.92–0.97]	0.97 [0.95–0.98]
**rMSSD, ms**	0.96 [0.94–0.97]	0.93 [0.89–0.95]	0.94 [0.90–0.96]
**pNN50, %**	0.91 [0.85–0.94]	0.86 [0.79–0.91]	0.86 [0.78–0.92]
**LF nu**	0.97 [0.95–0.98]	0.96 [0.94–0.98]	0.94 [0.90–0.96]
**HF nu**	0.97 [0.94–0.98]	0.98 [0.96–0.99]	0.97 [0.94–0.98]
**LF/HF**	0.95 [0.91–0.97]	0.95 [0.92–0.97]	0.98 [0.96–0.99]

Abbreviations: HRV, heart rate variability, 6MW, six-min walk test, SDNN, standard deviation of all R-R intervals, rMSSD, root mean square successive difference between R-R intervals, and pNN50, percentage of successive differences in the R-R interval of which the absolute values exceeds 50-ms, LF nu, low-frequency normalized unit, HF nu, high-frequency normalized unit, LF/HF, low and high-frequency ratio

## Discussion

To the best of our knowledge, the present study is the first to investigate the reproducibility of short-term HRV analysis using simultaneously Holter and PolarS810i and to examine the reliability of PolarS810i HRV analysis based on Holter in patients with moderate HF during the 6MW. The present study validated Polar in the short-term HRV analysis in this specific population of HF subjects with NYHA functional class II at rest. There was also good agreement between Polar and Holter on the HRV measures during the 6MW, despite a small effect of elevated HR (lower R-R intervals) on the absolute differences between the two devices, Holter and Polar.

Minimum 256 R-R intervals for HRV analysis were registered as recommended [1], in the three conditions (at rest, during the 6MW and during recovery) using both Holter and PolarS810i simultaneously. Low values of vagal indices and high values of sympathetic modulation were found in stable patients with moderate HF in all three conditions. These findings are consistent with other studies using portable ECGs and heart rate monitors [8, 24, 25]. The present study showed that short-term HRV indices are stable and reproducible using Holter and PolarS810i at rest because, as expected, the capturing and editing of stationary signals facilitate HRV analysis in stable conditions. Good CCC and Bland-Altman plots also indicated that PolarS810i produces good agreements between short-term HRV results, based on simultaneous recordings, and ECG at the three phases: at rest, during the 6MW and during recovery.

The reproducibility of short-term HRV indices using heart rate monitors in two consecutive 6MW was investigated by Corrêa et al. [26] using very short recordings at standing rest (1-min) and at the last 2 min of the 6MW in 38 obese subjects (15 male) aged 65 yrs. Subjects showed a similar performance in the two 6MW (mean difference = 2%), and the HRV indices were reproducible in time and non-linear domains. The authors concluded that these HRV indices may be useful in quantifying the effects of interventions on the autonomic modulation of the heart rate, particularly during exercise. In the present study, patients with HF NYHA II walked 15 meters longer in the second 6MW, which may be attributed to the well-documented high inter-test variability, related to fluctuating motivation, fatigue, and learning effects [27–30]. However, the rMSSD data from 6MW recovery in the first test were not similar to the second test using both devices, Holter and Polar S810i. This result may be associated with the highly skewed distribution of time-domain variables, as suggested by others in studies with ECG recordings [31].

In the short-term HRV index determination using ECG, a review of the literature showed that the reproducibility of these measurements is heterogeneous [32]. For instance, Ponikowski et al. [31] used 5-, 10-, 20- and 40-min recordings at supine rest. Although R-R intervals were reproducible (8% variability), the authors found poor reproducibility (25–139% variability) particularly for pNN50 and SDNN in patients with chronic HF, which is possibly attributed to the skewed distribution of these indices. However, it should be considered that the reproducibility in Ponikowski and co-workers study was tested in two moments with a large interval in between them (7 to 56 days), which might have allowed for changes in the clinical conditions of the patients with chronic HF and in turn changed the HRV indices. In our study, the two 6MW were controlled and had an interval of 60 min in between them, which may have helped avoid possible bias, such as changes in the environment and in the patients’ clinical conditions.

The simplicity and low cost of heart rate monitors combined with the good interchangeability with the gold-standard ECG for HRV analyses are advantages that have been largely demonstrated in healthy subjects [10–13] but not female subjects [14]. However, the agreement of short-term HRV indices between heart rate monitors and ECGs, based on ECG is little known in patients with HF. In our study, short-term HRV indices were analysed using two devices simultaneously. The analysis of agreement between the two devices was possible because of the use of a Holter with a roll-over analysis, which allowed us to focus on rapid and transitory changes by dividing time intervals into 5-min periods. We believe that this process of analysis was responsible for the high level of concordance between the values observed with Holter and Polar S810i in patients with moderate HF.

In healthy subjects, Kingsley et al. [33] assessed reliability and compared R-R intervals obtained during an incremental cycle ergometer test in healthy subjects using ECG and PolarS810i with a good correlation between devices. They showed good to excellent correlations (0.93–0.99) between the devices. In the present study, we also found excellent reliability of HRV indices at rest, during the 6MW and during recovery in patients with HF. We used the 6MW as a non-pharmacologic intervention to produce sympathetic activation. Fortunately, the signals were stable during the exercise, and by dropping the first minute of the 6MW, we succeeded in having the minimum 256 intervals for HRV analysis in the last five minutes.

The present study had some limitations. Participants were predominantly males who were aged, on average, 59 years and taking beta-blockers. However, there are studies that show that HRV results during exercise are independent of age and sex [34]. Previous studies that evaluated the reproducibility between Polar and Holter in the analysis of HRV in healthy subjects showed overall good intra-class correlation but unsatisfactory results in women [14] in frequency-domain variables [15] and rMSSD [11]. The use of beta-blockers is associated with increase in cardiac parasympathetic activity, in normal persons [35], and enhances HRV in patients with coronary artery disease possibly reducing heart rate and/or counteracting the adverse effects of sympathetic activity [36]. However, beta-blocker medications do not affect sudden beat-to-beat changes in R-R intervals [37] and the subjects of our study were simultaneously assessed by Holter and Polar for HRV measures. Additionally, we aimed to implement a real life scenario in which patients with stable HF take beta-blockers and other medications in their prescription. Another limitation is that, the 24-hour registration of the HRV can´t be represented by only 5 minutes analysis. However, several studies have validated the short-term HRV analysis in healthy subjects under controlled conditions ([10–17], Table 5). This study, which investigated the validity of Polar for HRV analysis in the specific population of HF patients, showed high agreement between all variables assessed simultaneously by Holter and Polar, which allowed us to assume the interchangeability of these methods in the evaluation of HRV in specific high-risk subjects. As suggested by others, the agreement that may be considered sufficient for the interchangeable use of two methods should have a 95% CI of > 0.75 [38]. Although there are intrinsic differences between Holter and Polar in signal acquisition, studies have validated Polar for short-term HRV analysis with good accuracy compared with the gold-standard method ECG in healthy subjects [10–17]. It is reasonable to expect that the quality of the signal acquisition during movement would be affected by transitory loss of signal and other factors. As pointed out before, Polar has important limitation for studies with patients with structural cardiac changes as it does not register ECG signals. However, in some way, this limitation seemed to be overcome in the HF population NYHA II. The present study validated Polar for HRV analysis during a sub-maximal functional capacity test can be considered simple and an important issue for future studies to assess the short-term response of beat-to-beat variations and, possibly, the response of the autonomic nervous system to specific interventions (for example, exercise) in HF patients NYHA class II. Although we have demonstrated through the linear regression model a small bias of proportionality in the correlation of the RR interval between the two devices (β = -0.010), this effect was small in magnitude. For instance, each reduction of 100 ms in the R-R interval was associated with an increase in one ms in the absolute difference between Holter and Polar measures. In this sense, besides the routine use of clinical variables and the walking distance registered in the 6MWT, the short-term HRV analysis during the 6MW can be another important tool for the health assessment of highly complex patients.

**Table 5:** Reliability between short-term ECG and portable heart rate monitors for HRV measurements in healthy subject.

Authors	N healthy subjects Age (years)	HRV recording	Statistical analysis	ECG results Mean (SD) or Median (IQR)	PolarS810i results Mean (SD) or Median (IQR)	ECG and Polar Agreement	Authors conclusion on agreement
**Barbosa et al.** [10]	30 male subjects Mean age = 21 yrs	**ECG and PolarRS800** 5 min—Supine	Pearson or Spearman correlation	SDNN = 58.8 (23.3)	SDNN = 57.1 (23.3)	SDNN = 0.99	For all variables
				rMSSD = 48.9 (26.5)	rMSSD = 47.4 (24.9)	rMSSD = 0.99	
				LF nu = 51.3 (14.2)	LF nu = 50.2 (12.8)	LF nu = 0.95	
				HFnu = 48.7 (14.2)	HF nu = 49.8 (12.8)	HF nu = 0.87	
				LF/HF = 1.35 (1.3)	LF/HF = 1.16 (0.7)	LF/HF = 0.91	
**Gamelin et al.** [11]	18 male subjects Mean age = 27 yrs	**ECG and Polar S810i** 5 min—Supine	Pearson or Spearman correlation	SDNN = 50.2 (18.8)	SDNN = 50.1 (18.8)	SDNN = 0.99	For all variables
				rMSSD = 46.7 (23.0)	rMSSD = 46.5 (24.0)	rMSSD = 0.99	
				pNN50 = 26.2 (21.0)	pNN50 = 25.9 (21.0)	pNN50 = 0.99	
				LF nu = 44.9 (22.0)	LF nu = 45.0 (22.9)	LF nu = 0.99	
				HF nu = 55.0 (23.0)	HF nu = 55.0 (22.9)	HF nu = 0.99	
				LF/HF = 1.2 (1.2)	LF/HF = 1.3 (1.2)	LF/HF = 0.99	
**Radespiel-Troger et al.** [12]	36 subjects Mean age = 27 yrs (22 male)	**ECG and Polar** 3 min—Sitting	Pearson or Spearman correlation	HR = 79 (11)	HR = 79 (12)	HR = 0.99	For all variables
				SDNN = 95.3 (44.0)	SDNN = 95.3 (43.0)	SDNN = 0.99	
				rMSSD = 58.0 (33.4)	rMSSD = 57.5 (33.0)	rMSSD = 0.99	
**Vanderlei et al.** [13]	15 male subjects Mean age = 21 yrs	**ECG and PolarS810i** 5 min—Supine	Intraclass correlation	rMSSD = 29.5 (3.3)	rMSSD = 29.7 (3.3)	rMSSD = 0.99	For all variables
				pNN50 = 52.8 (4.1)	pNN50 = 53.1 (4.2)	pNN50 = 0.99	
				LF nu = 60.5 (3.8)	LF nu = 61.7 (3.6)	LF nu = 0.97	
				HF nu = 39.5 (3.8)	HF nu = 8.3 (3.6)	HF nu = 0.97	
				LF/HF = 1.9 (0.3)	LF/HF = 1.9 (0.2)	LF/HF = 0.98	
**Wállen et al.** [14]	341 subjects Mean age = 52 yrs (139 male)	**ECG and PolarRS800** 5 min—Supine	Intraclass correlations	SDNN = 39.7 (37.7–41.7)	SDNN = 40.9 (38.8–43.2)	SDNN = 0.84	With limitations in female subjects
				rMSSD = 25.1 (23.4–26.9)	rMSSD = 25.2(23.6–26.9)	rMSSD = 0.93	
**Nunan et al.** [15]	33 subjects Median age = 34 yrs (19 male) Median age = 48 yrs (14 female)	**ECG and PolarS810i** 5 min—Supine	Intraclass correlation Data with log transformation	Mean R-R_log_ = 979.4 (176.9)	Mean R-R_log_ = 980.6 (178.6)	Mean R-R = 0.99	Only for time domain variables and LF/HF
				SDNN_log_ = 4.0 (0.5)	SDNN_log_ = 4.1 (0.5)	SDNN = 0.87	
				rMSSD_log_ = 3.7 (0.7)	rMSSD_log_ = 3.7 (0.6)	rMSSD = 0.88	
				LF nu_log_ = 59.0 (17.8)	LF nu_log_ = 62.5 (14.5)	LF nu = 0.75	
				HF nu_log_ = 41.0 (17.7)	HF nu_log_ = 37.5 (14.5)	HF nu = 0.72	
				LF/HF_log_ = 2.1 (2.1)	LF/HF_log_ = 2.2 (1.9)	LF/HF = 0.90	
**Porto et al.** [16]	25 subjects Mean age = 26 yrs (16 male)	**ECG and PolarS810i** 5 min—Supine	Limits of agreement Absolute Difference	Mean R-R = 949.0 (141.0)	Mean R-R = 951.0 (151.0)	Mean R-R = -6.4 to 2.7	For all variables
				SDNN = 61.2 (31.2)	SDNN = 60.9 (32.7)	SDNN = -1.7 to 2.3	
				rMSSD = 60.4 (35.7)	rMSSD = 59.6 (36.5)	rMSSD = not shown	
				pNN50 = 30.1 (32.3)	pNN50 = 32.3 (20.9)	pNN50 = -5.5 to 1.1	
**Weippert et al.** [17]	19 male subjects Mean age = 24 yrs	**ECG and PolarS810i** 3 min—Supine	Intraclass correlations	LF nu_log_ = 0.6 (0.2)	LF nu_log_ = 0.7 (0.2)	LF nu = 0.95	For all variables
				HF nu_log_ = 0.3 (0.1)	HF nu_log_ = 0.3 (0.1)	HF nu = 0.98	

## Conclusion

This study validates this portable, low-cost, easy-to-operate, and low-complexity heart rate monitor for the study of HRV at rest, during the 6MW and during recovery in patients with HF; it can thus be explored as a useful tool in future prospective interventional studies.

## Supporting Information

S1 File Raw data for each patient. (PDF)

## References

[1] Task Force of the European Society of Cardiology and the North American Society of Pacing and Electrophysiology. Heart-rate variability: standards of measurement, physiological interpretation and clinical use. Circulation. 1996; 93:1043–1065 8598068

[2] Stein PK, Rich MW, Rottman JN, Kleiger RE. Stability of index of heart rate variability in patients with congestive heart failure. Am Heart J. 1995; 129:975–981 7732987 10.1016/0002-8703(95)90119-1

[3] Van-Hoogenhuyze D, Weinstein N, Martin GJ, Weiss JS, Schaad JW, Sahyoni N, et al Reproducibility and relation to mean heart rate of heart rate variability in normal subjects and in patients with congestive heart failure secondary to coronary artery disease. Am J Cardiol. 1991; 68:1668–1676 1746470 10.1016/0002-9149(91)90327-h

[4] Nolan J, Batin PD, Andrews R, Lindsay SJ, Brooksby P, Mullen M, et al Prospective study of heart rate variability and mortality in chronic heart failure: results of the United Kingdom heart failure evaluation and assessment of risk trial (UK-Heart). Circulation. 1998; 98:1510–1516 9769304 10.1161/01.cir.98.15.1510

[5] Ponikowski P, Anker SD, Chua TP, Szelemej R, Piepoli M, Adamapoulos S, et al Depressed heart rate variability as an independent predictor of death in chronic congestive heart failure secondary to ischemic or idiopathic dilated cardiomyopathy. Am J Cardiol. 1997; 79:1645–1650 9202356 10.1016/s0002-9149(97)00215-4

[6] Bilchick KC, Fetics B, Djoukeng R, Fisher SG, Fletcher RD, Singh SN, et al Prognostic value of heart rate variability in chronic congestive heart failure (veterans affairs’ survival trial of antiarrhythmic therapy in congestive heart failure). Am J Cardiol. 2002; 90:24–28 12088774 10.1016/s0002-9149(02)02380-9

[7] Shah SA, Kambur T, Chan C, Herrington DM, Liu K, Shah SJ Relation of short-term heart rate variability to incident heart failure (from the multi-ethnic study of atherosclerosis). Am J Cardiol. 2013; 112:533–540 10.1016/j.amjcard.2013.04.018 23683953 PMC3735865

[8] LaRovere MT, Pinna GD, Maestri R, Mortara A, Copomolla S, Febo O, et al Short-term heart rate variability strongly predicts sudden cardiac death in chronic heart failure patients. Circulation. 2003; 107:565–570 12566367 10.1161/01.cir.0000047275.25795.17

[9] Lucreziotti S, Gavazzi A, Scelsi L, Inserra C, Klersy C, Campana C, et al Five-minute recording of heart rate variability in severe chronic heart failure: correlates with right ventricular function and prognostic implications. Am Heart J. 2000; 139:1088–1095 10.1067/mhj.2000.106168 10827392

[10] Barbosa MPCR, Silva NT, Azevedo FM, Pastre CM, Vanderlei LCM Comparison of Polar RS800G3 heart rate monitor with Polar S810i and electrocardiogram to obtain the series of RR intervals and analysis of heart rate variability at rest. Clin Physiol Funct Imaging. 2014; 10.1111/cpf.12203 25348547

[11] Gamelin FX, Berthoin S, Bosquet L Validty of the Polar S810 heart rate monitor to measure R-R intervals at rest. Med Sci Sports Exerc. 2006; 38:887–893 10.1249/01.mss.0000218135.79476.9c 16672842

[12] Radespiel-Troger M, Rauh R, Mahlke C, Gottschalk T, Muck-Weymann M Agreement of two different methods for measurement of heart rate variability. Clin Auton Res. 2003; 13:99–102 10.1007/s10286-003-0085-7 12720094

[13] Vanderlei LCM, Silva RA, Pastre CM, Azevedo FM, Godoy MF Comparison of the Polar S810i monitor and the ECG for the analysis of heart rate variability in the time and frequency domains. Braz J Med Biol Res. 2008; 41:854–859 18853042 10.1590/s0100-879x2008005000039

[14] Wallén MB, Hasson D, Theorel T, Canlon B, Osika W Possibilities and limitations of the polar RS800 in measuring heart rate variability at rest. Eur J Appl Physiol. 2012; 112:1153–1165 10.1007/s00421-011-2079-9 21766225

[15] Nunan D, Jakovljevic DG, Donovan G, Hodges LD, Sandercock GRH, Brodie DA Levels of agreement for RR intervals and short-term heart rate variability obtained from the Polar S810 and an alternative system. Eur J Appl Physiol. 2008; 103:529–537 10.1007/s00421-008-0742-6 18427831

[16] Porto LGG, Junqueira LF Comparison of time domain short-term heart interval variability analysis using a wrist-worn heart rate monitor and the conventional electrocardiogram. PACE. 2009; 32:43–51 10.1111/j.1540-8159.2009.02175.x 19140912

[17] Weippert M, Kumar M, Kreuzfeld S, Arndt D, Rieger A, Stoll R Comparison of three mobile device for measuring R-R intervals and heart rate variability: Polar S810i, Suuntot6 and an ambulatory ECG system. Eur J Appl Physiol. 2010; 109:779–786 10.1007/s00421-010-1415-9 20225081

[18] Chobanian AV, Bakris GL, Black HR, Cushman WC, Green LA, Izzo JL Jr, et al The national high blood pressure education program coordinating committee. Seventh report of the Joit National Committee of Prevetion, Detection, Evaluation and Treatment of High Blood Pressure, Hypertension. 2003; 42:1206–1252 10.1161/01.HYP.0000107251.49515.c2 14656957

[19] American Diabetes Association, Diagnosis and classification of diabetes. Diabetes Care. 2015; 38:S8–S16 10.2337/dc15-S005 25537714

[20] Craig CL, Marshal AL, Sjostrom M, Bauman AE, Booth ML, Ainsworth BE, et al International physical activity questionnaire: 12-country reliability and validity. Med Sci Sports Exerc. 2003; 35:1381–1395 10.1249/01.MSS.0000078924.61453.FB 12900694

[21] ATS statement: guidelines for the six-minute walk test. Am J Resp Crit Care Med. 2002; 166:111–117 10.1164/ajrccm.166.1.at1102 12091180

[22] Lin LIK A concordance coefficient to evaluate reproducibility. Biometrics. 1989; 45:255–268 2720055

[23] Bland JM, Altman DG Statistical methods for assessing agreement between two methods of clinical measurements. Lancet. 1986; 1:307–310 2868172

[24] Jiang W, Hathaway WR, McNutry S, Larsen RL, Hansley KL, Zhang Y, et al Ability of heart rate variability to predict prognosis in patients with advanced congestive heart failure. Am J Cardiol. 1997; 80:808–811 9315600 10.1016/s0002-9149(97)00526-2

[25] Caruso FCR, Arena R, Mendes RG, Reis MS, Papa V, Borghi-Silva A Heart rate autonomic responses during deep breathing and walking in hospitalised patients with chronic heart failure. Disabil Rehabil. 2011; 33:751–757 10.3109/09638288.2010.511420 20731562

[26] Corrêa FR, Guerra RLF, Dourado VZ Reliability of heart rate variability analysis during submaximal exercise in subjects aged 60 years and older. J Resp Cardiov Phy Ther. 2012; 1:37–43

[27] Wu G, Sanderson B, Bittner V The 6-minute walk test: how important is the learning effect? Am Heart J. 2003; 146:129–133 10.1016/S0002-8703(03)00119-4 12851620

[28] Hamilton DM, Haennel RG Validity and reliability of the 6-minute walk test in a cardiac rehabilitation population. J Cardiopulm Rehabil. 2000; 20:156–164 10860197 10.1097/00008483-200005000-00003

[29] Kervio G, Carre F, Ville NS Reliability and intensity of the six-minute walk test in healthy elderly subjects. Med Sci Sports Exerc. 2003; 35:169–174 10.1249/01.MSS.0000043545.02712.A7 12544651

[30] Hernandes NA, Wouters EF, Meijer K, Annegarn J, Pitta F, Spruit MA Reproducibility of 6-minute walking test in patients with COPD Eur Respir J. 2011; 38:261–267 10.1183/09031936.00142010 21177838

[31] Ponikowski P, Piepoli M, Amadi AA, Chua TP, Harrington D, Volerrani M, et al Reproducibility of heart rate variability measures in patients with chronic heart failure Clin Sci (Lond). 1996; 91:391–398 8983864 10.1042/cs0910391

[32] Sandercock GRH, Bromley PD, Brodie DA The reliability of short-term measurements of heart rate variability. Int J Cardiol. 2005; 103:238–247 10.1016/j.ijcard.2004.09.013 16098384

[33] Kingsley M, Lewis MJ, Marson RE Comparison of PolarS810i and an ambulatory ECG system for RR interval measurement during progressive exercise. Int J Sports Med. 2005; 26:39–44 10.1055/s-2004-817878 15643533

[34] Corrêa FR, Silva Alves MA, Bianchim MS, Aquino AC, Guerra RLF, Dourado VZ Heart rate variability during 6-min walk test in adults aged 40 years and older. Int J Sports Med. 2013; 34:111–115 10.1055/s-0032-1321888 22972244

[35] Cook JR, Bigger JT, Kleiger RE, Fleiss JL, Steinman RC, Rolnitzky LM. Effect of atenolol and diltiazem on heart period variability in normal persons. J Am Coll Cardiol. 1991; 17:480–484. 1991906 10.1016/s0735-1097(10)80119-6

[36] Niemela MJ, Airaksinen KEJ, Huikuri HV. Effect of beta-blockade on heart-rate-variability in patients with coronary-artery disease. J Am Coll Cardiol. 1994; 23(6):1370–1377. 8176095 10.1016/0735-1097(94)90379-4

[37] Malfatto G, Facchini M, Sala L, Branzi G, Bragato R, Leonetti G Effects of cardiac rehabilitation and beta-blocker therapy on heart rate variability after first acute myocardial infarction. Am J Cardiol. 1998; 81:834–840 9555771 10.1016/s0002-9149(98)00021-6

[38] Sandercock GRH, Shelton C, Bromley P, Brodie DA Agreement between three commercially available instruments for measuring short-term heart rate variability. Physiol Meas. 2004; 25:1115–1124 15535178 10.1088/0967-3334/25/5/003

