# Supplementary material for: Reproducibility for Heart Rate Variability Analysis during 6-Min Walk Test in Patients with Heart Failure and Agreement between Devices
Source: PLoS One. 2016 Dec 9;11(12):e0167407. doi: 10.1371/journal.pone.0167407 (PMC5147870; doi:10.1371/journal.pone.0167407)
Supplement: S1 File — (PDF) [file pone.0167407.s001.pdf]

number	AGE	gender	BMI	NYHA	FEVE	Etiol IC	TABAG	packyears	HAS	DM	DLP	Hipotiroi	IAM	AA	AC	ACEI	AIIA	AP	BB	VC	D	DIU	ST	QRS1
1	55	1	25,7	2	33	1	2	30	1	0	1	0	1	0	0	0	0	1	1	1	0	1	1	100
2	65	1	22,8	2	37	1	2	29	1	1	1	0	1	0	0	1	0	1	1	0	0	1	1	120
3	60	1	22,1	2	43	1	2	40	1	1	1	0	1	0	0	0	1	1	1	1	1	1	1	110
4	64	2	22,2	2	40	1	0	0	1	0	1	1	1	0	0	0	1	1	0	0	1	1	1	90
5	65	1	23,9	2	30	1	2	30	1	1	2	1	1	0	0	1	0	1	1	1	1	1	1	110
6	62	1	29,8	2	30	2	2	13	1	1	1	0	0	1	0	1	0	1	1	1	1	1	1	120
7	46	1	21,3	2	54	1	2	15	1	0	1	0	1	0	0	0	0	1	1	1	0	1	1	100
8	60	2	40,6	2	35	1	2	19	1	1	1	0	1	0	0	1	0	1	1	0	0	1	1	180
9	51	1	20,4	2	17	1	2	17	1	0	1	0	1	0	0	1	0	1	1	0	0	1	1	120
10	51	1	24	2	29	1	2	19	1	1	1	0	1	0	0	1	0	1	1	0	0	1	1	160
11	60	1	25,9	2	29	1	2	2	1	0	1	0	1	0	0	1	0	1	1	1	0	1	1	140
12	65	1	23,6	2	42	1	0	0	1	0	1	0	1	0	1	0	1	1	1	1	0	1	1	100
13	56	1	17,5	2	28	1	2	10	1	1	1	0	1	1	0	1	0	1	1	1	0	1	1	180
14	62	1	28,2	2	35	1	2	19	1	0	1	0	1	0	0	0	0	1	1	1	0	1	1	220
15	65	1	22,5	2	32	1	2	38	1	0	1	0	1	0	0	0	0	1	1	1	0	1	1	120
16	63	1	21,8	2	37	1	2	17	1	0	1	0	1	0	0	1	0	1	1	1	0	1	1	110
17	65	1	32	2	37	1	2	17	1	0	1	0	1	0	0	1	0	1	1	1	0	1	1	140
18	61	2	15,8	2	41	3	2	2	1	0	1	0	0	0	0	0	1	1	1	0	0	1	1	90
19	59	1	31	2	26	3	2	60	1	0	1	0	0	0	0	1	0	1	1	1	0	1	1	200
20	64	1	32,5	2	37	1	0	0	1	1	1	0	1	0	0	1	0	1	1	1	1	1	1	100
21	64	1	23,7	2	42	1	0	0	1	0	1	0	1	0	0	1	0	1	1	1	0	1	1	100
22	47	1	30,8	2	36	2	0	0	1	1	1	0	0	0	0	1	0	1	1	0	0	1	1	110
23	61	1	26,9	2	55	1	2	3	1	1	1	0	1	0	0	0	0	1	1	1	1	1	1	90
24	56	1	20,6	2	28	1	2	27	1	1	1	0	1	0	0	0	1	1	1	1	1	1	1	100
25	49	1	16,9	2	45	3	0	0	1	0	1	0	0	0	0	1	0	1	1	0	0	1	1	110
26	54	1	19,7	2	46	1	0	0	1	1	1	0	1	0	0	1	0	1	1	1	0	1	1	190
27	59	1	33,4	2	30	1	2	44	1	1	1	0	1	0	0	1	0	1	1	1	1	1	1	120
28	64	2	27,3	2	34	1	0	0	1	0	1	1	1	0	1	1	0	1	1	1	0	1	1	120
29	48	1	29	2	30	1	2	0,5	1	0	1	0	1	1	0	1	0	1	1	1	0	1	1	120
30	65	1	20,2	2	35	1	0	0	1	1	1	0	1	0	0	1	0	1	1	1	0	1	1	110
31	42	1	30	2	25	1	2	1	1	0	1	0	1	0	0	0	1	1	1	1	1	1	1	100
32	65	1	25	2	28	1	0	0	1	1	1	0	1	1	1	1	0	1	1	1	1	1	1	120

33	52	1	30,3	2	42	1	2	6	1	1	1	0	1	0	0	1	0	1	1	1	0	1	1	100
34	65	1	22,3	2	34	2	2	10	1	0	1	0	0	1	0	1	0	1	1	1	1	1	1	200
35	58	1	18,5	2	41	1	2	30	1	0	1	0	1	0	1	1	0	0	1	1	0	1	1	120
36	55	1	20,4	2	33	1	2	30	1	0	1	0	1	1	0	1	0	1	1	1	0	1	1	180
37	60	1	27,5	2	30	1	2	39	1	1	1	0	1	0	0	0	1	1	1	1	0	1	1	150
38	65	1	25	2	36	1	2	24	1	1	1	1	1	1	1	0	1	0	1	1	0	0	1	120
39	62	2	26,2	2	31	1	2	30	1	1	1	0	1	0	0	0	1	1	1	1	0	1	1	110
40	51	1	14,4	2	33	1	0	0	1	0	1	0	1	0	1	0	0	0	1	1	0	1	1	140
41	64	2	22,2	2	48	1	2	16	1	1	1	0	1	0	0	1	0	1	1	1	0	1	1	110
42	64	2	18,8	2	52	2	0	0	1	0	1	1	0	1	0	0	0	1	1	0	0	1	1	100
43	64	1	20,6	2	24	1	0	0	1	0	1	0	1	0	0	1	0	1	1	1	0	1	1	140
44	60	1	24,3	2	29	2	2	31,5	1	0	1	0	0	0	0	1	0	1	1	0	0	1	1	110
45	65	2	25,4	2	39	1	2	15,8	1	0	1	0	1	0	0	1	0	1	1	1	0	1	1	160
46	54	2	22,8	2	38	3	2	52	1	0	1	0	0	1	0	0	1	1	1	0	0	1	1	110
47	60	2	22,1	2	34	1	0	0	1	1	1	0	1	0	0	1	0	1	1	1	1	1	1	140
48	57	1	26,9	2	28	3	2	22,5	1	0	1	0	0	0	1	1	0	0	1	0	1	1	1	120
49	65	1	34,2	2	40	2	2	43,5	1	0	1	0	0	0	1	0	1	0	1	0	0	1	1	150
50	49	2	19,2	2	30	2	2	17	1	0	1	0	0	0	0	0	1	1	1	1	0	1	1	170
	**58,86**		**24,56**		**35,36**			**16,40**																**128,60**
	**6,22**		**5,23**		**7,73**			**16,16**																**32,45**

QRS2	HFCR1	PFCR1	HFCR2	PFCR2	HFC62	PFC62	HFCRC1	PFCRC1	HFCRC2	PFCRC2	HRRMR1	PRRMR1	HRRMR2	PRRMR2	HRRM62
100	62	62	66	66	128	129	74	72	73	75	969,2	961,5	910,4	909,4	470,2
120	51	51	60	59	80	79	62	61	62	61	1172,1	1168,0	1005,4	1002,0	754,0
110	63	63	66	66	89	88	66	66	68	69	957,4	950,3	910,6	916,0	670,0
90	65	65	65	65	111	112	91	90	75	75	934,4	929,8	930,1	925,4	543,0
110	62	62	59	59	81	82	62	60	59	59	975,4	964,8	1014,4	1014,2	744,5
120	65	65	67	67	91	90	71	71	76	78	936,1	929,2	888,6	888,5	658,0
100	50	50	50	51	72	71	52	51	51	51	1213,0	1212,0	1251,0	1159,0	833,0
180	59	58	58	58	88	88	71	69	64	64	1040,1	1036,2	1046,0	1039,0	680,0
120	59	58	61	61	89	90	61	61	68	67	1039,4	1023,5	997,2	980,6	674,9
160	63	63	76	75	106	108	75	74	81	79	957,2	952,4	791,7	806,0	566,2
140	67	67	67	67	83	83	69	68	67	67	893,4	890,7	900,4	893,0	725,0
110	61	62	58	58	77	77	63	62	57	58	977,0	972,2	1040,1	1033,4	781,0
170	51	50	48	47	78	79	50	50	49	49	1197,0	1190,4	1272,0	1268,1	777,0
220	58	58	62	62	81	80	63	63	64	64	1035,0	1033,4	960,6	957,7	739,0
120	57	56	57	56	75	76	61	60	57	57	1070,4	1068,5	1071,9	1068,4	798,0
120	65	65	70	71	106	105	75	74	70	71	916,9	913,0	852,4	842,0	566,5
130	68	68	65	67	72	73	69	66	71	70	888,2	878,0	917,1	890,0	834,0
90	62	62	55	55	90	91	61	61	60	60	973,8	973,5	1091,2	1091,5	664,0
180	69	69	69	69	88	87	76	75	72	71	866,4	866,0	867,0	871,0	685,0
100	87	87	90	90	120	120	96	95	109	110	687,4	687,2	665,8	663,5	498,0
100	63	62	60	59	96	95	63	63	63	63	964,0	958,9	1010,0	1003,4	625,0
120	56	51	55	56	78	79	54	51	58	56	1062,0	1039,5	1083,0	1062,8	763,9
100	77	75	74	73	111	112	83	82	74	73	802,8	780,8	824,4	816,2	540,0
100	68	68	69	69	83	84	69	69	69	69	881,0	879,7	874,5	871,4	727,6
100	66	65	63	63	95	96	70	69	67	67	917,5	915,0	956,8	957,3	634,0
190	71	71	67	67	98	97	80	76	77	76	882,8	851,5	896,1	890,0	616,0
130	66	66	67	66	92	92	71	70	69	69	907,1	906,0	902,0	897,3	649,0
120	58	57	58	58	76	77	56	56	55	53	1044,6	1044,3	1041,8	1026,6	795,0
120	63	62	62	61	93	94	63	62	63	63	960,7	950,0	978,0	968,2	642,3
110	56	56	54	53	76	77	57	55	55	57	1088,4	1091,4	1120,6	1122,1	791,0
110	67	67	61	61	76	77	62	62	65	64	904,0	900,6	972,3	970,6	784,5
130	59	59	62	61	82	82	67	66	67	67	1024,2	1013,8	974,6	973,7	735,5

90	66	66	53	53	77	77	64	64	54	54	908,9	904,2	1135,0	1135,3	783,0
200	61	60	57	56	92	92	71	72	63	60	1011,0	1013,0	1058,0	1050,0	652,2
120	70	70	77	77	116	116	81	81	87	86	861,2	857,2	777,6	776,9	517,0
180	57	57	56	56	73	74	56	56	56	56	1056,2	1056,9	1069,4	1067,8	810,8
140	60	60	60	60	109	108	69	69	69	69	991,7	989,2	1009,2	1004,2	549,0
120	72	71	67	67	79	80	68	68	66	66	842,6	839,4	896,8	889,3	763,2
110	57	57	56	56	110	111	65	62	67	65	1055,2	1050,1	1083,0	1076,5	541,0
140	62	62	61	61	101	101	68	70	68	67	988,4	981,4	985,0	974,2	598,3
110	96	94	95	95	114	114	110	109	105	104	628,8	628,4	629,4	630,6	526,3
100	55	55	53	53	74	73	53	53	56	56	1125,6	1123,0	1136,2	1136,4	811,2
140	61	59	57	59	75	76	60	58	60	57	1021,1	1017,9	1051,9	1011,1	802,0
110	51	51	52	52	85	85	59	57	57	56	1167,8	1146,5	1161,8	1162,6	705,0
160	56	56	59	58	81	82	57	57	59	59	1065,1	1038,6	1039,6	1032,2	742,0
110	52	52	48	48	61	61	57	56	58	55	1151,6	1146,3	1242,2	1242,7	993,0
140	58	55	57	56	83	83	61	60	61	60	1032,0	1029,2	1052,2	1040,0	725,0
120	58	57	78	78	117	119	63	62	68	67	755,6	746,6	760,0	773,1	515,0
160	58	57	57	58	96	97	63	62	58	57	1226,0	1221,0	1052,9	1043,0	625,0
160	65	63	53	53	75	74	70	70	73	73	1140,2	1139,7	1142,9	1137,0	800,9
**128,60**	**62,58**	**62,04**	**62,34**	**62,24**	**89,58**	**89,86**	**67,16**	**66,32**	**66,40**	**65,98**	**983,4**	**977,2**	**986,0**	**979,2**	**688,5**
**30,77**	**8,39**	**8,38**	**9,42**	**9,43**	**15,26**	**15,36**	**11,16**	**11,12**	**11,43**	**11,57**	**126,1**	**126,2**	**136,1**	**131,6**	**111,1**

PRRM62	HRRMRC1	PRRMRC1	HRRMRC2	PRRMRC2	HSDNNR1	PSDNNR1	HSDNNR2	PSDNNR2	HSDNN62	PSDNN62	HSDNNRC1
464,1	829,8	826,5	826,2	794,2	26,6	26,3	34,0	30,2	29,0	26,5	24,9
756,8	968,6	947,7	975,2	977,2	42,2	38,5	48,3	48,0	29,0	30,1	40,6
678,0	853,0	836,8	878,6	867,0	41,0	38,0	35,8	39,1	27,9	24,4	41,2
533,5	704,9	661,4	797,8	784,5	38,4	36,7	45,0	42,1	31,0	28,7	49,9
735,5	994,2	985,9	1019,7	1010,4	34,0	33,5	34,2	34,3	26,7	24,0	30,8
663,8	845,0	844,1	790,0	766,8	13,8	11,8	9,1	9,0	20,0	19,9	12,6
845,0	1173,0	1169,2	1204,0	1201,0	47,2	48,9	43,0	41,1	31,0	29,7	50,6
678,2	894,4	890,0	929,0	926,6	21,0	21,2	27,0	26,0	24,3	22,0	26,7
667,8	979,2	970,2	879,2	898,5	35,8	33,5	37,8	35,0	18,0	19,6	39,8
557,1	807,4	801,3	735,0	758,4	51,2	49,8	40,4	34,4	17,7	18,9	29,9
723,6	891,6	887,8	894,4	894,9	20,6	20,2	14,6	13,5	27,0	26,5	23,0
775,1	975,2	973,5	1046,8	1043,1	25,8	25,3	30,2	28,0	34,0	32,1	33,0
763,9	1193,0	1194,3	1221,8	1219,6	32,8	32,4	33,6	27,9	31,0	27,5	33,4
746,2	960,6	959,8	943,0	939,9	17,2	16,4	15,6	14,3	28,0	25,9	14,4
789,9	999,2	991,3	1061,4	1058,6	21,4	20,8	19,9	18,6	30,9	29,9	27,2
573,2	818,8	814,2	785,5	781,8	30,0	28,4	53,6	50,4	26,0	24,2	46,6
817,1	910,6	902,6	856,8	841,6	30,6	28,6	32,8	29,8	21,1	19,8	34,4
661,7	990,5	987,0	998,2	994,3	14,6	14,3	14,6	13,7	23,2	20,2	19,4
689,8	798,8	785,8	845,8	831,8	13,0	12,5	14,6	12,1	21,0	21,9	28,8
489,9	629,8	623,2	546,0	545,3	19,2	18,1	18,5	17,6	11,0	11,2	18,4
631,6	950,0	949,9	962,0	957,0	40,0	38,8	23,9	22,0	23,0	21,4	26,0
760,3	1075,6	1073,4	1045,4	1036,8	47,2	45,5	51,3	50,1	37,4	38,6	40,8
538,1	734,2	726,0	824,4	814,0	34,4	32,8	31,1	30,8	15,0	13,8	29,9
712,7	875,8	874,4	870,4	866,8	18,2	17,5	21,5	21,0	20,5	18,7	20,9
621,8	867,0	862,5	893,4	888,6	21,0	17,5	15,2	14,6	35,0	33,1	20,8
620,0	788,1	772,0	788,2	778,1	37,4	37,6	33,6	27,6	33,6	29,8	40,6
651,0	765,2	703,6	862,8	863,8	20,0	18,9	22,3	21,0	15,0	14,5	26,9
791,7	1073,4	1070,8	1128,4	1121,6	18,7	14,8	35,6	33,9	27,3	28,1	29,7
636,1	972,9	967,2	955,2	953,0	47,8	43,5	30,9	26,2	25,1	23,7	34,8
778,0	1092,6	1011,1	1096,2	1099,2	37,0	34,9	23,2	20,0	27,3	25,9	27,4
777,3	965,8	965,6	950,4	941,3	33,4	31,9	36,4	34,6	16,6	15,1	25,0
728,5	912,2	911,5	900,8	896,7	26,4	22,9	20,2	17,3	18,5	18,6	17,0

784,0	938,9	938,4	1107,0	1102,4	54,2	53,3	27,0	26,9	17,0	15,4	24,2
649,0	966,6	968,0	994,7	929,8	56,0	53,3	57,0	54,6	22,0	19,7	37,4
519,2	743,2	739,9	700,9	698,2	23,8	23,5	19,8	19,9	35,0	33,7	21,2
811,2	1089,5	1087,6	1077,2	1072,6	16,6	15,8	21,2	19,7	18,8	17,0	35,1
553,7	866,1	864,8	870,3	868,6	53,4	52,2	43,8	42,2	29,0	30,9	21,5
751,5	886,6	881,0	914,6	910,9	20,2	18,1	42,2	37,6	25,0	23,7	22,8
537,2	968,9	960,0	930,8	922,3	30,2	28,4	33,9	31,3	29,7	26,1	46,2
597,0	881,0	890,1	890,0	885,0	27,1	24,2	35,0	32,1	35,9	32,0	39,0
535,1	559,6	556,2	559,0	577,2	14,0	13,3	13,0	11,3	16,0	17,8	40,9
826,6	1126,7	1125,2	1136,9	1136,6	19,8	19,3	18,2	16,8	19,2	17,3	19,8
807,9	1031,3	1028,8	1052,1	1043,5	16,1	14,5	18,9	17,4	15,7	13,4	10,8
701,3	1058,2	1051,4	1079,6	1063,2	39,0	39,2	30,8	29,2	25,1	22,4	36,2
735,8	1042,6	1040,0	1019,3	1014,0	39,5	35,0	40,0	38,7	26,0	26,9	22,5
989,0	1048,8	1035,9	1056,4	1036,0	16,6	16,1	19,6	18,3	17,0	15,8	32,6
722,1	1007,9	1004,6	999,0	967,9	26,5	24,4	19,0	18,6	19,0	17,0	24,2
504,0	784,5	779,7	728,0	715,9	19,8	17,2	22,1	20,0	29,4	26,0	18,0
618,0	1081,0	1078,0	903,4	899,0	34,0	32,3	27,1	25,0	18,5	17,0	26,0
811,2	853,8	851,9	1153,0	1145,0	29,2	27,7	27,0	24,3	17,4	15,6	41,3
**686,2**	**924,5**	**916,4**	**933,7**	**926,8**	**29,9**	**28,4**	**29,3**	**27,4**	**24,4**	**23,0**	**29,7**
**111,4**	**134,0**	**136,3**	**146,7**	**145,3**	**11,9**	**11,8**	**11,5**	**11,1**	**6,5**	**6,3**	**9,8**

PSDNNRC1	HSDNNRC2	PSDNNRC2	HRMSSDR1	PRMSSDR1	HRMSSDR2	PRMSSDR2	HRMSSD62	PRMSSD62	HRMSSDRC1	PRMSSDRC1
23,4	27,8	26,6	23,0	21,3	17,0	15,8	14,3	13,0	13,8	12,3
40,3	29,8	26,6	17,9	16,7	18,0	16,3	14,9	12,8	15,4	15,2
40,0	30,6	34,1	21,6	19,4	27,4	24,1	19,0	18,0	31,6	29,0
47,0	45,0	42,0	23,4	19,5	20,2	18,7	13,0	12,0	14,6	10,9
29,0	25,0	23,0	22,2	21,4	24,8	23,6	16,2	14,4	17,4	17,7
13,4	15,9	12,4	12,6	12,5	8,4	8,5	7,1	6,4	10,0	8,8
49,0	52,0	49,8	11,8	12,3	15,0	12,2	11,4	12,5	17,4	15,2
24,2	25,7	24,8	13,2	15,7	23,4	22,2	16,0	17,2	24,5	23,4
38,9	36,8	37,9	17,6	16,8	25,0	24,1	11,0	13,5	31,0	29,5
29,4	38,1	40,4	20,8	19,4	26,5	25,8	16,0	14,5	21,0	18,4
22,7	15,8	13,4	9,2	7,8	12,2	10,9	8,9	6,3	9,0	7,9
30,2	23,6	22,3	21,6	19,8	26,0	24,1	12,8	11,1	19,0	18,4
28,2	41,5	39,2	23,1	21,9	25,0	23,2	16,4	14,4	19,7	17,1
13,3	18,8	17,9	14,8	13,4	13,8	11,7	11,0	12,4	11,6	10,4
26,7	20,0	19,9	16,2	15,7	9,0	8,6	18,0	16,3	7,0	6,7
42,6	35,2	32,1	15,6	15,1	17,8	16,4	14,0	11,9	17,0	14,6
33,6	38,3	35,8	24,4	21,9	21,5	19,9	11,1	12,4	14,0	12,9
17,7	14,4	12,4	12,2	11,4	19,6	17,7	15,0	12,8	15,4	15,7
29,7	13,8	13,6	11,8	12,4	12,8	12,0	19,0	15,9	15,5	13,4
17,2	19,2	17,9	13,6	11,9	8,0	6,9	9,0	7,4	10,5	7,9
23,9	31,0	29,4	27,1	26,2	15,0	14,1	17,0	15,4	14,0	12,0
39,4	51,4	49,4	35,0	33,0	26,4	25,5	14,0	12,9	32,4	31,1
28,6	26,2	24,0	9,8	9,9	10,6	9,5	12,0	10,4	8,0	7,0
18,9	30,6	28,7	9,0	8,3	10,4	9,7	7,4	6,5	11,0	10,4
20,0	25,1	22,8	15,0	13,5	15,4	12,9	16,4	14,9	13,8	13,0
39,5	27,6	27,7	20,2	17,7	19,1	18,0	12,8	11,0	16,6	16,6
23,9	18,2	15,7	14,4	12,9	15,0	14,7	11,6	12,4	15,0	11,5
26,4	45,2	42,9	18,2	16,8	24,0	23,8	15,9	13,8	24,0	23,0
31,1	35,2	33,3	16,8	12,4	20,0	17,8	8,1	6,9	22,0	17,4
25,2	29,6	31,2	14,9	13,4	9,6	10,2	13,0	14,7	12,0	12,5
20,5	44,6	44,2	9,8	9,4	17,0	15,8	11,7	12,8	15,2	14,7
13,9	17,0	15,9	14,8	12,2	19,5	18,2	14,0	12,1	7,0	5,7

19,5	43,1	42,0	15,9	13,4	15,0	13,0	12,6	9,7	13,2	10,7
41,2	53,0	47,9	5,3	4,3	7,6	6,2	10,6	11,0	9,2	8,1
18,8	24,0	21,9	13,8	9,9	9,0	7,7	12,9	11,8	10,0	9,8
32,6	42,2	37,0	9,0	8,6	11,8	10,1	12,0	11,0	16,0	14,6
18,9	18,8	17,4	25,0	24,1	30,1	28,1	18,0	16,7	15,3	12,0
21,7	40,5	36,7	15,5	14,0	20,4	18,4	7,0	6,9	15,9	14,4
38,4	46,0	42,1	21.9	18,0	22,5	20,0	17,4	18,2	21,0	18,4
40,2	39,2	37,0	14,0	12,9	12,9	11,0	13,3	14,1	18,4	17,0
37,6	25,1	24,6	10,0	7,9	12,4	10,1	11,0	11,9	12,5	11,2
19,9	19,6	18,9	11,0	10,3	11,0	9,7	12,0	13,1	13,1	12,8
9,9	18,2	18,4	15,0	14,6	12,0	11,5	9,7	10,2	12,2	10,3
37,7	35,3	33,4	15,8	14,6	18,8	18,0	11,0	10,4	13,6	12,0
19,6	35,7	32,0	25,0	20,0	24,1	21,5	18,0	16,9	22,0	18,3
30,9	54,8	53,8	8,4	7,5	8,0	6,7	12,0	11,0	11,0	8,9
23,2	24,0	21,9	22,0	17,4	23,7	23,0	16,0	15,1	21,0	19,2
16,3	17,9	14,0	12,0	10,2	17,0	14,9	19,0	16,1	11,4	8,6
23,4	32,0	29,3	21,0	17,9	12,2	10,9	12,0	13,2	14,0	12,9
39,9	34,0	31,9	27,0	22,0	19,0	15,9	12,0	10,9	17,0	15,4
**28,0**	**31,0**	**29,4**	**16,7**	**15,2**	**17,2**	**15,8**	**13,3**	**12,5**	**15,9**	**14,3**
**9,7**	**11,3**	**11,0**	**6,0**	**5,5**	**6,1**	**5,9**	**3,2**	**3,0**	**5,8**	**5,6**

HRMSSDRC2	PRMSSDRC2	HPNN50R1	PPNN50R1	HPNN50R2	PPNN50R2	HPNN5062	PPNN5062	HPNN50RC1	PPNN50RC1	HPNN50RC2
16,0	13,9	1,6	1,0	0,2	0,0	0,3	0,0	0,3	0,2	0,3
17,0	15,1	2,0	1,8	1,7	1,3	1,8	1,0	6,4	4,2	1,9
20,2	19,4	1,8	1,6	2,9	1,2	1,9	0,3	6,8	5,9	2,6
24,0	19,8	2,9	1,8	1,8	0,0	1,2	0,5	0,9	0,0	1,6
17,6	16,9	0,5	0,6	2,0	1,7	0,4	0,2	0,0	0,0	0,7
13,3	11,2	0,0	0,0	0,0	0,0	0,6	0,0	0,0	0,0	0,3
18,4	15,1	4,7	4,5	3,9	3,8	2,7	2,0	3,9	3,4	3,8
30,0	29,9	0,0	0,0	0,0	0,0	0,0	0,0	0,0	0,0	0,0
34,0	31,2	5,7	4,0	4,6	4,9	0,3	0,0	2,5	1,8	2,3
20,8	19,4	2,7	2,2	1,7	1,3	1,6	0,0	0,6	0,5	1,4
8,4	7,8	0,6	0,0	0,8	0,0	0,0	0,0	0,3	0,0	0,1
21,0	21,3	0,3	0,0	2,4	2,0	3,3	2,6	1,2	0,6	2,0
23,6	22,5	1,0	0,4	1,0	0,4	1,9	0,0	2,1	0,0	2,0
14,1	12,9	0,0	0,0	0,2	0,0	0,0	0,0	0,0	0,0	0,1
8,8	7,9	0,4	0,4	0,0	0,0	1,8	0,0	0,0	0,0	0,0
15,0	14,2	1,5	1,0	2,6	0,3	1,2	0,5	1,9	1,3	1,1
13,8	11,2	2,0	1,8	1,2	0,8	0,5	0,0	0,5	0,6	0,4
15,6	14,2	0,0	0,0	1,5	0,4	0,4	0,0	0,0	0,0	0,3
15,2	14,4	0,0	0,0	0,0	0,0			0,0	0,0	0,0
8,0	8,1	0,4	0,0	0,0	0,0	0,8	0,0	0,0	0,0	0,0
15,0	13,4	0,9	0,8	0,3	0,0	1,8	1,5	0,3	0,0	0,7
29,8	27,2	5,6	6,1	4,7	4,4	1,6	0,5	5,1	4,4	4,1
11,2	9,2	0,0	0,0	0,2	0,0	0,3	0,2	0,0	0,0	0,0
14,2	13,3	0,0	0,0	0,0	0,0	0,5	0,0	0,0	0,0	0,0
14,0	12,1	0,2	0,3	0,2	0,0	1,7	0,2	0,0	0,0	0,0
13,4	10,4	0,8	0,0	0,9	0,0	1,4	1,1	0,2	0,0	0,7
14,4	12,9	0,0	0,0	0,6	0,0	0,8	0,0	0,7	0,7	0,2
18,8	17,6	0,3	0,0	2,8	2,1	1,0	0,0	2,1	1,2	0,3
19,0	16,9	2,0	0,0	0,3	0,3	0,5	0,0	1,4	0,0	0,9
15,0	11,5	0,4	0,0	0,0	0,0	3,9	3,1	0,1	0,0	1,4
13,0	11,1	0,0	0,0	1,3	0,3	0,3	0,0	0,0	0,0	0,2
12,2	11,8	0,5	0,3	1,4	0,0	1,3	0,2	0,0	0,0	0,0

12,0	12,2	0,3	0,0	0,8	0,0	1,2	0,0	0,5	0,0	0,0
15,5	13,4	0,0	0,0	0,0	0,0	1,0	0,0	0,9	0,2	1,9
7,8	6,1	0,0	0,0	0,0	0,0	2,0	0,0	0,5	0,0	0,0
16,6	15,6	0,0	0,0	0,1	0,0	0,0	0,0	0,0	0,0	0,0
16,0	10,9	5,0	4,9	7,9	6,7	1,6	0,0	0,0	0,0	0,2
27,0	24,6	0,2	0,0	1,2	1,2	0,2	0,0	0,0	0,0	3,5
25,0	24,4	4,5	3,0	1,7	2,0	1,5	1,0	0,6	0,0	3,9
21,0	20,4	1,2	0,8	0,0	0,0	1,0	0,8	3,1	2,9	2,7
14,5	14,2	0,0	0,0	0,6	0,0	0,0	0,0	1,5	0,4	2,0
12,8	11,5	0,0	0,0	0,0	0,0	0,0	0,0	0,1	0,0	0,0
14,1	12,9	0,0	0,0	0,0	0,0	0,0	0,0	0,0	0,0	0,0
12,2	11,8	3,1	2,8	4,3	3,5	1,6	0,0	3,6	3,2	2,9
17,0	15,0	3,9	3,7	3,0	3,8	1,5	0,2	1,9	2,0	1,6
29,2	27,8	0,0	0,0	0,0	0,0	0,0	0,0	0,0	0,0	2,8
22,1	20,0	0,0	0,0	0,0	0,0	0,8	0,4	1,3	0,4	1,3
12,0	11,0	0,3	0,0	1,5	0,7	2,6	1,9	0,1	0,0	0,0
20,0	17,3	0,0	0,0	2,0	1,6	1,4	0,0	1,9	0,0	0,0
26,0	21,1	6,6	5,4	6,6	4,9	3,0	2,1	2,3	1,5	5,0
**17,3**	**15,7**	**1,3**	**1,0**	**1,4**	**1,0**	**1,1**	**0,4**	**1,1**	**0,7**	**1,1**
**6,0**	**5,8**	**1,8**	**1,6**	**1,8**	**1,6**	**0,9**	**0,8**	**1,6**	**1,4**	**1,3**

PPNN50RC2	HLFR1	PLFR1	HLFR2	PLFR2	HLF62	PLF62	HLFRC1	PLFRC1	HLFRC2	PLFRC2	HHFR1	PHFR1	HHFR2	PHFR2	HHF62
0,0	63,8	61,3	67,9	69,8	78,8	77,5	73,8	74,1	73,1	73,9	36,2	38,7	32,2	29,9	21,2
1,6	60,6	56,7	75,7	75,3	74,5	73,7	77,7	71,8	35,1	64,9	39,2	41,2	24,7	24,3	25,5
0,5	58,6	56,5	54,3	58,6	63,9	63,3	64,5	62,9	57,1	60,1	49,4	43,5	45,7	49,9	36,1
0,0	85,9	89,9	87,5	90,3	76,7	79,5	90,0	88,7	61,6	63,9	14,1	17,3	14,6	17,5	36,1
0,7	63,5	62,0	57,2	54,4	64,5	61,2	52,3	50,4	43,1	46,7	38,0	35,9	45,6	42,7	38,4
0,0	67,2	65,9	79,3	78,5	90,9	94,2	83,1	78,1	80,1	83,3	34,1	39,6	20,7	21,2	26,7
3,8	60,4	59,6	56,8	59,9	89,8	82,3	57,5	60,5	52,9	54,6	40,4	43,7	43,2	45,4	30,8
0,0	75,6	73,9	62,9	65,1	70,6	69,3	69,9	66,6	68,3	70,0	41,5	38,9	37,1	40,5	26,1
1,0	82,1	80,8	74,4	76,6	90,1	93,3	62,4	62,1	55,5	58,1	18,8	17,8	25,6	23,4	18,0
1,2	73,9	69,1	68,9	67,4	68,2	64,3	79,0	80,2	76,4	79,9	26,9	24,8	34,8	32,5	31,8
0,0	84,2	81,9	70,9	68,5	76,2	74,1	72,4	70,3	77,1	75,7	14,2	15,3	29,1	31,4	23,8
2,0	88,3	88,8	77,8	76,9	84,0	82,7	72,8	72,2	76,9	79,2	11,7	11,1	22,2	25,9	16,0
1,6	63,9	65,0	72,8	72,3	51,5	50,8	66,3	64,0	62,6	64,8	36,1	34,9	27,2	27,7	48,5
0,0	83,1	79,5	77,5	75,7	68,6	70,6	71,4	69,6	76,7	73,2	26,9	27,6	22,5	21,1	31,4
0,0	63,5	62,5	65,0	64,4	67,6	62,3	90,9	87,5	48,0	53,1	36,5	37,4	35,0	35,4	32,4
0,3	65,7	67,9	91,5	88,9	89,4	91,8	79,2	80,6	70,1	71,2	44,3	42,0	8,5	11,0	12,5
0,0	99,8	97,9	84,2	79,9	99,4	98,7	80,1	78,2	100,1	95,3	12,4	10,0	23,2	20,1	13,6
0,3	54,1	51,4	62,4	60,3	78,9	81,6	56,5	56,3	71,7	74,9	45,9	47,7	32,8	35,1	27,4
0,0	89,1	86,8	84,3	86,8	58,5	57,1	83,5	82,4	90,0	90,9	15,1	13,2	13,8	12,9	41,5
0,0	82,2	82,5	83,0	84,8	79,0	79,1	70,6	71,2	89,0	92,8	17,8	17,5	17,0	15,2	21,0
0,3	73,5	71,6	58,6	57,2	63,3	61,2	74,9	73,4	67,9	68,7	26,5	28,4	41,4	42,8	36,7
3,8	75,4	74,5	89,2	90,2	92,9	88,7	53,9	54,2	71,2	71,3	25,5	24,5	14,4	12,9	9,9
0,0	89,3	87,2	92,8	94,4	76,9	71,1	92,7	93,5	85,2	84,4	13,8	18,8	8,3	7,6	26,1
0,0	60,2	58,9	79,8	77,1	82,2	81,3	90,9	88,8	79,3	81,7	40,8	42,1	21,2	22,7	23,4
0,0	67,4	51,5	50,7	54,5	72,6	76,3	49,7	42,0	53,6	53,8	48,5	45,6	49,3	45,4	22,4
0,0	61,8	59,0	65,0	65,3	76,1	78,9	76,9	80,3	64,6	68,4	41,0	38,5	35,2	34,7	23,2
0,0	54,6	51,4	54,2	57,4	76,0	75,0	58,5	56,6	69,0	69,9	45,4	48,4	45,8	42,3	23,2
0,4	72,8	73,9	70,4	69,4	76,2	74,2	58,3	59,7	72,4	68,9	37,2	35,9	46,6	40,4	23,8
0,0	77,7	71,1	70,8	76,4	87,7	89,6	45,9	49,2	72,0	72,2	22,3	28,9	29,2	23,6	12,3
0,7	63,6	67,9	70,9	75,4	87,0	85,7	80,4	87,8	87,0	84,1	36,4	32,9	29,1	32,1	20,7
0,3	77,7	81,2	89,6	84,2	78,5	77,6	57,2	55,7	78,5	80,3	22,8	18,8	10,4	15,7	21,5
0,0	61,4	58,4	57,9	60,6	69,7	62,2	72,2	72,3	73,0	74,6	38,6	41,6	40,1	39,4	

0,0	68,6	68,2	79,6	81,0	73,3	75,7	73,3	72,5	76,6	79,4	31,4	31,5	20,4	18,9	26,7
1,2	87,7	93,6	71,3	68,5	80,2	81,3	74,5	70,1	73,7	69,7	12,3	16,6	28,7	31,3	19,8
0,0	84,2	85,5	84,9	88,7	83,9	80,6	83,9	85,7	77,7	79,1	15,8	14,4	15,1	11,3	20,1
0,0	77,7	70,5	85,7	86,2	93,0	89,3	82,5	81,2	87,5	87,0	22,3	29,4	14,3	13,8	10,7
0,0	64,1	66,8	57,8	62,7	68,4	65,3	74,4	78,6	55,6	52,6	35,9	33,2	42,2	37,2	31,6
2,9	55,2	58,2	74,1	75,2	84,3	81,8	59,6	63,7	61,2	60,3	44,8	41,8	25,9	24,8	15,7
3,6	77,8	73,6	77,5	71,5	73,5	75,4	75,5	72,2	71,9	69,5	22,2	26,4	22,5	24,4	26,5
2,8	89,6	91,5	83,6	85,9	89,3	89,0	79,4	81,3	83,5	89,3	10,4	8,4	16,4	19,2	10,7
0,0	75,9	77,3	69,7	69,9	85,0	88,1	84,1	85,7	75,9	78,3	24,3	22,6	30,3	29,9	17,0
0,0	54,9	55,1	92,7	88,8	59,4	59,5	77,6	74,9	61,3	60,8	45,1	41,7	13,3	12,2	40,6
0,0	62,3	60,9	72,4	74,8	84,0	82,1	64,4	65,4	62,9	64,7	41,1	40,6	34,9	31,7	28,6
2,5	77,8	78,5	25,2	21,5	83,6	81,7	89,4	92,8	15,6	17,2	25,2	21,5	19,1	22,4	16,4
1,3	71,9	77,6	82,7	80,7	74,4	75,2	79,5	81,1	87,7	85,2	28,4	22,4	17,3	21,2	25,6
2,4	95,3	93,6	88,3	85,4	90,5	92,3	50,1	54,4	68,6	72,8	14,7	16,3	13,5	14,6	12,4
0,0	96,5	93,5	75,4	80,6	78,3	76,1	75,0	73,1	92,2	92,5	8,8	6,5	24,6	19,8	21,7
0,0	71,2	69,1	63,7	62,5	85,1	83,7	75,4	72,6	90,3	93,7	28,8	25,9	36,3	37,5	14,9
0,0	73,9	70,3	67,5	70,1	81,4	83,5	67,8	70,7	75,9	77,2	21,2	18,9	32,5	35,4	22,0
3,5	84,5	81,2	80,3	85,4	87,7	91,0	67,9	65,4	67,1	65,7	13,4	11,9	33,4	29,9	38,0
**0,8**	**73,4**	**72,2**	**72,7**	**73,1**	**78,3**	**77,6**	**72,0**	**71,7**	**70,5**	**72,0**	**28,9**	**28,7**	**27,3**	**27,2**	**24,5**
**1,2**	**12,0**	**12,6**	**13,1**	**13,0**	**10,1**	**10,8**	**11,8**	**12,0**	**15,3**	**14,1**	**12,0**	**11,9**	**11,2**	**10,7**	**9,0**

PHF62	HHFRC1	PHFRC1	HHFRC2	PHFRC2	HLFHFR1	PLFHFR1	HLFHFR2	PLFHFR2	HLFHF62	PLFHF62	HLFHFRC1	PLFHFRC1	HLFHFRC2
22,5	26,2	25,9	26,9	26,1	1,8	1,6	2,1	2,3	3,7	3,4	2,9	2,9	2,7
26,2	22,3	25,9	35,1	33,2	1,5	1,4	3,5	3,1	2,9	2,8	3,5	2,8	1,8
36,7	45,5	39,1	37,5	39,9	1,2	1,3	1,2	1,2	1,8	1,7	1,2	1,6	1,5
38,4	14,3	16,9	38,4	36,1	6,1	5,2	6,2	5,2	2,2	2,1	6,3	5,3	1,6
36,6	47,7	49,5	56,9	53,2	1,9	1,6	1,4	1,3	1,7	1,7	1,1	1,1	0,8
25,3	21,9	19,8	25,0	26,6	1,9	1,7	3,8	3,7	3,4	3,7	3,6	4,2	3,2
27,5	42,5	43,5	49,8	47,0	1,6	1,4	1,3	1,3	2,9	3,0	1,4	1,9	1,2
28,0	30,0	25,1	31,7	29,9	1,8	1,9	1,7	2,1	2,7	2,5	2,5	2,1	2,2
16,7	46,0	47,8	43,1	45,9	4,3	4,7	2,9	3,3	5,0	5,6	1,4	1,3	1,3
35,7	22,0	20,1	19,7	19,9	2,8	2,7	1,9	2,1	2,1	1,8	3,6	4,0	4,0
25,9	29,7	27,6	32,8	34,3	5,8	5,5	2,4	2,2	3,2	2,9	2,4	2,6	3,0
17,3	27,2	27,8	23,1	20,7	7,5	8,0	3,5	3,0	5,3	4,8	2,7	2,6	3,3
47,0	36,0	33,9	39,4	37,9	1,8	1,9	2,7	2,6	1,1	1,1	1,8	2,0	2,0
29,4	28,6	25,7	23,3	26,6	3,1	2,9	3,5	3,6	2,2	2,4	2,5	2,7	3,3
37,3	11,9	9,0	42,4	46,9	1,7	1,7	1,9	1,8	2,1	1,7	7,4	10,1	1,0
10,6	17,8	19,3	30,1	28,8	1,5	1,6	10,7	8,0	7,2	8,7	4,4	4,2	2,3
14,2	20,2	21,8	5,2	4,6	8,0	10,5	3,6	4,0	7,3	6,9	4,0	4,0	19,2
28,3	43,5	42,2	25,0	25,9	1,2	1,1	1,9	1,7	2,9	2,4	1,3	1,3	2,9
42,7	16,8	17,2	10,9	8,8	5,9	6,6	6,1	6,7	1,4	1,3	5,0	4,8	8,3
20,6	29,4	28,8	21,0	17,2	4,6	4,7	4,9	5,6	3,8	3,9	2,4	2,4	4,2
38,8	25,1	26,6	32,1	31,3	2,8	2,5	1,4	1,3	1,7	1,6	3,0	2,8	2,1
10,9	46,0	45,7	28,8	28,7	2,9	3,1	6,2	7,0	9,3	8,1	1,2	1,2	2,5
28,8	10,1	9,7	14,8	15,6	5,3	4,6	12,3	12,4	2,9	2,5	9,2	9,6	5,7
20,6	15,2	16,1	20,7	18,3	1,5	1,4	2,3	3,4	3,3	3,9	6,2	5,7	3,9
23,7	58,0	50,1	46,4	46,1	1,2	1,5	1,1	1,2	3,4	3,2	0,8	0,9	1,2
21,0	22,2	19,7	28,4	31,4	1,4	1,6	1,8	1,9	3,3	3,8	3,5	4,0	2,3
24,4	41,5	46,3	31,0	32,4	1,2	0,9	0,9	1,4	3,3	3,1	1,4	1,2	2,2
20,9	41,7	40,1	27,6	31,3	2,2	2,5	1,5	1,7	3,2	3,6	1,4	1,5	2,6
11,4	55,0	50,8	28,0	27,8	3,8	2,5	2,4	3,2	7,1	8,6	0,8	0,9	2,6
19,3	12,2	15,4	13,0	15,9	1,7	2,1	2,4	2,3	6,7	4,2	7,2	5,9	6,7
19,6	42,2	44,2	21,5	19,6	3,7	4,3	8,6	5,7	3,7	4,2	1,4	1,3	3,7
30,3	27,8	27,7	36,9	25,3	1,6	1,4	1,4	1,6	2,3	1,7	2,6	2,6	2,7

24,1	27,5	26,4	23,4	20,6	2,2	2,2	3,9	4,3	2,7	3,1	2,6	2,8	3,3
18,7	32,9	36,7	26,3	29,2	7,1	6,0	2,5	2,2	4,1	4,4	2,3	1,9	2,8
18,4	16,1	14,2	22,3	20,8	5,3	6,0	5,7	7,5	4,2	4,4	5,2	6,0	3,5
11,5	17,5	18,8	12,5	12,9	3,7	2,4	6,0	6,3	8,7	7,8	4,7	4,3	7,0
34,6	25,6	21,4	44,4	47,3	1,8	2,0	1,4	1,7	2,2	1,9	2,9	3,7	1,3
18,1	40,4	36,8	38,8	39,7	1,2	1,4	3,2	3,1	5,4	4,5	1,5	1,8	1,6
24,6	24,5	27,6	28,1	30,4	3,5	2,9	3,4	3,0	2,8	3,1	3,1	2,7	2,6
11,0	20,6	19,4	16,5	14,9	8,6	10,9	5,1	5,7	8,3	8,1	3,8	4,2	5,1
16,7	19,2	18,2	19,4	21,7	3,1	3,4	2,3	2,3	5,0	5,2	4,2	4,9	3,8
39,9	22,4	25,2	38,7	38,1	1,2	1,3	7,0	7,1	1,5	1,5	3,5	3,0	0,6
27,0	40,9	39,6	49,1	45,1	1,6	1,5	2,1	2,4	2,9	3,0	1,6	1,7	1,3
18,4	15,6	17,2	16,2	13,9	3,1	3,7	4,5	3,9	5,1	4,4	5,5	5,4	5,2
24,4	20,5	18,9	12,3	14,1	2,5	3,5	5,0	4,0	2,9	3,1	3,9	4,3	7,1
11,8	49,9	44,8	21,4	21,1	6,5	5,7	6,5	5,8	7,4	7,7	1,0	1,2	3,2
20,4	25,0	26,9	7,8	7,4	11,0	14,4	3,5	4,2	3,6	3,7	3,0	2,7	11,8
16,3	9,3	11,0	9,7	7,9	2,5	2,7	1,8	1,7	5,7	5,1	7,8	6,9	9,3
20,5	16,4	19,1	26,1	22,8	3,5	3,7	2,1	1,9	3,7	4,1	4,1	3,7	2,9
36,7	22,4	19,9	44,2	47,1	6,1	5,4	2,4	2,9	2,3	2,5	2,9	3,4	1,5
**24,6**	**28,5**	**28,0**	**28,1**	**27,8**	**3,4**	**3,5**	**3,6**	**3,6**	**3,9**	**3,8**	**3,3**	**3,3**	**3,6**
**9,2**	**12,5**	**11,8**	**11,9**	**12,1**	**2,3**	**2,8**	**2,4**	**2,3**	**2,0**	**2,0**	**2,0**	**2,0**	**3,2**

PLFHFRC2	DP1	TC6M%P1	DP2	TC6M%P2	SF36P	SF36M	MLHF	IPAQ
2,8	621,6	97,9	619,8	97,6	46,3	39,3	19	2
2,0	390,0	79,9	418,2	85,0	38,6	53,2	26	2
1,5	480,0	94,0	481,2	94,3	46,4	51,5	6	3
1,8	460,2	97,7	490,8	104,2	44,8	57,1	25	2
0,9	450,0	92,0	481,2	98,5	39,9	40,8	23	2
3,1	543,6	103,0	547,2	103,8	50,4	52,2	22	2
1,2	480,0	76,7	483,6	77,2	36,4	24,1	62	2
2,2	345,6	80,0	363,0	84,9	35,5	42,3	20	2
1,3	431,4	87,0	439,8	89,7	33,0	50,0	46	3
4,0	491,4	85,7	510,0	88,0	40,2	64,1	26	3
3,1	489,6	88,0	487,8	87,9	41,3	52,4	28	2
3,8	433,8	91,0	450,0	94,0	27,0	36,7	42	2
1,8	511,2	101,0	510,0	100,0	22,9	26,9	26	2
2,8	415,2	69,9	412,2	69,3	55,0	52,6	5	3
1,1	319,2	56,0	320,0	56,3	49,6	36,0	18	2
2,5	486,0	106,0	508,8	111,0	41,1	51,8	32	2
20,7	407,4	82,0	421,2	85,0	26,3	50,9	47	2
3,0	546,6	69,3	546,0	69,2	57,0	38,1	22	2
10,3	426,6	75,8	450,0	79,9	36,9	53,0	28	3
5,4	472,2	100,0	491,4	104,0	49,3	55,8	25	2
2,2	529,2	91,7	602,4	104,4	45,9	61,4	12	2
2,5	420,0	63,7	450,0	69,2	37,3	56,8	30	2
5,4	481,2	81,7	491,4	83,4	46,1	34,7	37	3
4,5	420,0	82,0	430,8	84,0	44,3	37,7	34	2
1,2	570,0	107,0	592,2	111,5	42,2	29,9	40	2
2,2	510,0	82,0	540,0	86,0	37,9	45,4	25	2
2,0	522,0	105,0	516,0	104,5	36,7	28,3	55	2
2,2	450,0	94,7	470,4	99,0	37,5	52,4	53	2
2,6	483,0	72,5	488,4	73,3	26,4	34,6	34	2
5,2	391,2	77,7	396,0	78,7	42,4	50,6	22	3
4,1	382,8	51,1	418,2	65,0	28,5	54,3	60	2
2,9	366,0	64,7	475,8	84,1	38,5	65,9	25	2

number
AGE
sex
imc
NYHA
FEVE
ETIOL IC
TABAG
packyears
HAS
DM
DLP
Hipotireoid
IAM
AA
AC
ACEI
AIIA
AP
BB
CV
D
DIU
ST
QRS1
QRS2
HFCR1
PFCR1
HFCR2
PFCR2
HFC62
PFC62
HFCRC1

3,9	447,6	83,1	453,6	84,2	36,4	54,6	29	2
2,4	506,4	89,5	511,2	90,4	38,2	53,9	39	3
3,8	588,0	102,0	600,0	104,0	39,6	49,1	28	2
6,7	417,0	74,9	450,0	80,5	25,1	44,2	54	3
1,1	565,5	114,0	570,0	115,6	30,3	29,5	29	2
1,5	591,0	111,7	600,0	113,4	28,3	33,7	20	2
2,3	459,0	101,0	465,0	102,7	18,1	56,3	31	2
6,0	510,0	78,7	511,8	79,0	41,0	49,5	23	2
3,7	472,2	98,9	472,0	98,7	26,9	26,9	37	2
0,6	444,0	87,9	450,0	89,0	40,3	46,9	21	2
1,4	469,8	87,9	514,2	96,3	64,4	48,1	25	2
6,5	619,8	100,4	634,8	102,9	48,0	55,8	20	2
6,1	463,8	97,1	430,8	90,2	30,8	41,4	9	2
3,6	510,0	97,0	500,4	95,1	33,0	66,1	24	3
12,5	477,0	95,9	481,0	96,8	31,6	39,7	45	2
11,9	510,0	102,0	552,0	110,8	51,2	60,2	11	3
3,4	476,4	97,0	510,0	104,0	33,5	62,4	32	2
1,4	450,0	78,5	451,8	78,8	38,5	54,5	20	2
**3,8**	**474,1**	**88,1**	**489,2**	**91,1**	**38,7**	**47,1**	**29,44**	**2,22**
**3,5**	**65,8**	**14,1**	**64,7**	**13,6**	**9,2**	**11,0**	**13,14**	**0,42**

PFCRC1
HFCRC2
PFCRC2
HRRMR1
PRRMR1
HRRMR2
PRRMR2
HRRM62
PRRM62
HRRMRC1
PRRMRC1
HRRMRC2
PRRMRC2
HSDNNR1
PSDNNR1
HSDNNR2
PSDNNR2
HSDNN62
PSDNN62
HSDNNRC1

enrollment
Idade
1= masculino/ 2 = feminino
Indice de massa corporea
New York Heart Associoation (2= classe funcional)
Fração de ejeção ventrículo esquerdo (%)
Etiologia da Insuficiência cardíaca (1 = isquêmica 2 = dilatada 3 = valvar)
Tabagismo (0 = não 1 = sim 2 = ex)
packyears = number of cigarettes x years /20
Hipertensão arterial sistemica (0 = não 1 = sim)
Diabetes mellitus (0 = não 1 = sim)
Dislipdemia (0 = não 1 = sim)
Hipotireoidismo (0 = não 1 = sim)
Infarto agudo do miocardio (0 = não 1 = sim)
Antiarritmico (0 = não 1 = sim)
Anticoagulante (0 = não 1 = sim)
Enzima inibidora da conversão da angiotensina (0 = não 1 = sim)
Antagonistas dos receptores da angiotensina (0 = não 1 = sim)
Antiplaquetário (0 = não 1 = sim)
Betabloqueador (0 = não 1 = sim)
Vasodilatador coronariano (0 = não 1 = sim)
Digitálico (0 = não 1 = sim)
Diurético (0 = não 1 = sim)
Estatinas (0 = não 1 = sim)
complexo QRS teste 1
complexo QRS teste 2
holter frequencia cardíaca de repouso teste 1
polar frequencia cardíaca de repouso teste 1
holter frequencia cardíaca de repouso teste 2
polar frequencia cardíaca de repouso teste 2
holter frequencia cardíaca 6 minutos
polar frequencia cardíaca 6 minutos
holter frequencia cardíaca de recuperação teste 1

PSDNNRC1	polar SDNN recuperação teste 1
HSDNNRC2	holter SDNN recuperação teste 2
PSDNNRC2	polar SDNN recuperação teste 2
HRMSSDR1	holter RMSSD repouso teste 1
PRMSSDR1	polar RMSSD repouso teste 1
HRMSSDR2	holter RMSSD repouso teste 2
PRMSSDR2	polar RMSSD repouso teste 2
HRMSSD62	holter RMSSD 6 minutos
PRMSSD62	polar RMSSD 6 minutos
HRMSSDRC1	holter RMSSD recuperação teste 1
PRMSSDRC1	polar RMSSD recuperação teste 1
HRMSSDRC2	holter RMSSD recuperação teste 2
PRMSSDRC2	polar RMSSD recuperação teste 2
HPNN50R1	holter PNN50 repouso teste 1
PPNN50R1	polar PNN50 repouso teste 1
HPNN50R2	holter PNN50 repouso teste 2
PPNN50R2	polar PNN50 repouso teste 2
HPNN5062	holter PNN50 6 minutos
PPNN5062	polar PNN50 6 minutos
HPNN50RC1	holter PNN50 recuperação teste 1
PPNN50RC1	polar PNN50 recuperação teste 1
HPNN50RC2	holter PNN50 recuperação teste 2
PPNN50RC2	polar PNN50 recuperação teste 2
HLFR1	holter LF repouso teste 1
PLFR1	polar LF repouso teste 1
HLFR2	holter LF repouso teste 2
PLFR2	polar LF repouso teste 2
HLF62	holter LF 6 minutos
PLF62	polar LF 6 minutos
HLFRC1	holter LF recuperação teste 1
PLFRC1	polar LF recuperação teste 1
HLFRC2	holter LF recuperação teste 2
PLFRC2	polar LF recuperação teste 2

DP1	distância pe
TC6M%P1	teste da ca
DP2	distância pe
TC6M%P2	teste da ca
SF36P	qualidade c
SF36M	qualidade c
MLHF	qualidade c
IPAC	questionári

polar frequencia cardíaca de recuperação teste 1
holter frequencia cardíaca de recuperação teste 2
polar frequencia cardíaca de recuperação teste 2
holter RR medio de repouso teste 1
polar RR medio de repouso teste 1
holter RR medio de repouso teste 2
polar RR medio de repouso teste 2
holter RR medio 6 minutos
polar RR medio 6 minutos
holter RR medio de recuperação teste 1
polar RR medio de recuperação teste 1
holter RR medio de recuperação teste 2
polar RR medio de recuperação teste 2
holter SDNN repouso teste1
polar SDNN repouso teste 1
holter SDNN repouso teste 2
polar SDNN repouso teste 2
holter SDNN 6 minutos
polar SDNN 6 minutos
holter SDNN recuperação teste 1

HHFR1	holter HF repouso teste 1
PHFR1	polar HF repouso teste 1
HHFR2	holter HF repouso teste 2
PHFR2	polar HF repouso teste 2
HHF62	holter HF 6 minutos
PHF62	polar HF 6 minutos
HHFRC1	holter HF recuperação teste 1
PHFRC1	polar HF recuperação teste 1
HHFRC2	holter HF recuperação teste 2
PHFRC2	polar HF recuperação teste 2
HLFHFR1	holter LFHF repouso teste 1
PLFHFR1	polar LFHF repouso teste 1
HLFHFR2	holter LFHF repouso teste 2
PLFHFR2	polar LFHF repouso teste 2
HLFHF62	holter LFHF 6 minutos
PLFHF62	polar LFHF 6 minutos
HLFHFRC1	holter LFHF recuperação teste 1
PLFHFRC1	polar LFHF recuperação teste 1
HLFHFRC2	holter LFHF recuperação teste 2
PLFHFRC2	polar LFHF recuperação teste 2

ercorrida teste 1
minhada dos seis minutos % predito teste 1
ercorrida teste 2
minhada dos seis minutos % predito teste 2
de vida short form 36 (dominio físico)
de vida short form 36 (dominio mental)
de vida minessota living with heart failure
o de atividade física
